# High-precision determination of nitrite, nitrate, phosphate, and silicate for the characterization of MOOS-4 certified reference material for nutrients in seawater

**DOI:** 10.1007/s00216-025-05928-7

**Published:** 2025-05-31

**Authors:** Enea Pagliano, Zuzana Gajdosechova

**Affiliations:** Metrology Research Center, National Research Council Canada, 1200 Montreal Road, Ottawa, ON K1A 0R6 Canada

**Keywords:** Certified reference materials, Nutrients in seawater, Nitrite, Nitrate, Phosphate, Silicate

## Abstract

High-precision determination of inorganic nutrients (i.e., nitrite, nitrate, phosphate, and silicate) in seawater is paramount for understanding variations in marine biogeochemical cycles. Historically, the lack of consistency between nutrient data sets has been the Achilles’ heel for large-scale oceanographic studies and the regular use of certified reference materials (CRMs) was identified as a solution to improve data quality. In this study, the preparation and certification of NRC MOOS-4 nearshore seawater CRM for nutrients are presented. The discussion is focused on the optimization of the analytical methods that were developed in decade-long research. On the one hand, the classical spectrophotometric methods were studied with attention to potential systematic biases not normally considered in routine analysis. On the other hand, higher-order (isotope dilution) methods with better performance characteristics were developed for accurate quantitation of nitrite, nitrate, and silicate. This rigorous method development work resulted in exceptional agreement between nutrient data obtained by traditional and modern analytical methodologies and allowed improvement of measurement uncertainties with respect to previous MOOS-3 CRM. As a result, MOOS-4 could be certified with expanded (*k* = 2) uncertainties between 0.8% and 2.6%, consistent with the requirements of the oceanographic community (i.e., *c*(NO_2_^−^) = 1.676 ± 0.013 µmol/L, *c*(NO_3_^−^) = 17.14 ± 0.44 µmol/L, *c*(PO_4_^3−^) = 2.835 ± 0.031 µmol/L, *c*(SiO_2_) = 7.16 ± 0.13 µmol/L). In line with the FAIR principles for scientific data management, all measurement data for value assignment and software used for data analysis was made available in the Electronic Supporting Material.

**Graphical Abstract:**

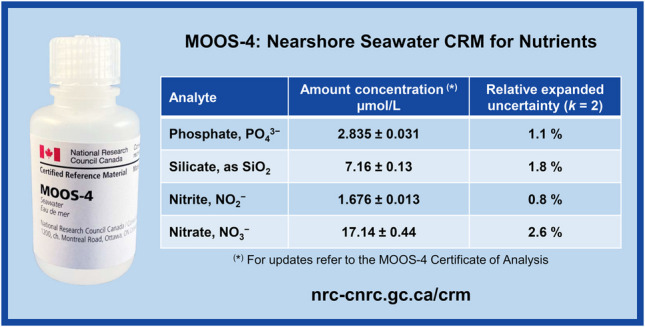

**Supplementary Information:**

The online version contains supplementary material available at 10.1007/s00216-025-05928-7.

## Introduction

Inorganic nutrients play an essential role in the regulation of marine primary production [1], influencing the carbon cycle [2], and serving as potential indicators for large-scale changes in marine biology [3]. Hence, their accurate determination is essential for monitoring the health of marine ecosystems, and sustaining fisheries [4]. Routine measurements of nutrients in seawater (i.e., nitrite, nitrate, phosphate, and silicate) are commonly performed using spectrophotometric methods based on continuous (segmented) flow analyzers which are simple instrumental apparatus suitable for at-sea analysis on shipboard [5, 6]. These procedures are based on classical wet chemistry for the formation of colored derivatives detectable by UV–vis [7, 8] and are suitable for research cruises which typically require processing hundreds of samples per day. In the past two decades, a great research effort has been devoted to further improve continuous flow nutrient analyzers leading to the development of lab-on-a-chip microfluidic devices which could be used as well for direct in situ analysis [9–12]. However, current methods’ advantages of automation, portability, green chemistry, and high sample throughput have come at the expense of measurement accuracy and precision. Systematic biases between nutrient data sets are not uncommon and can certainly be a limiting factor for data interpretation. In 2007, the Intergovernmental Panel on Climate Change (IPCC) has been one of the most authoritative voice to express concerns about the quality of nutrient data emphasizing “the lack of comparability between [nutrients] measurements from different laboratories at different time” [3]. Matrix effects, low specificity and sensitivity are common shortcomings limiting the performance of common methods for nutrients measurement in seawater [12, 13], especially at the low nanomolar concentration typical for oligotrophic waters [14, 15].

The oceanographic community has identified the routine use of certified reference materials (CRMs) for quality control as the necessary means to achieve the required level of consistency within global nutrient data [5] along with a sound evaluation of measurement uncertainty [10]. Example of CRMs available from National Metrology Institutes include the NRC MOOS-4 [16] and SALT-1 [17] and the three NMIJ/AIST CRMs 7601-a, 7602-a, and 7603-a [18]. Other suppliers include the Scripps Institute of Oceanography, Kanso Technos, the Korean Institute of Ocean Science and Technology (KIOST), and Eurofins Scientific, just to mention a few.

The development of nutrients CRMs relies on the availability of higher-order analytical methods for value assignment which can complement and effectively cross-validate the results returned by continuous flow analyzers and microfluidic devices. Methods developed *ad hoc* for CRMs characterization should promote high-precision, accuracy, and traceability, without the necessity of high sample throughput or in situ analysis [18–22].

This study describes the preparation of the NRC MOOS-4 CRM for nutrients in nearshore seawater and focuses on the analytical method development that allowed improving measurement uncertainties within the range required for oceanographic studies [5]. High-precision methods based on isotope dilution mass spectrometry (IDMS) were used for quantitation of nitrite, nitrate, and silicate [23], whereas spectrophotometry methods based on gravimetric samples preparation were used for nitrite, phosphate and silicate. The agreement between independent methodologies was excellent and the CRM could be certified within an expanded uncertainty (*k* = 2) between 0.8% and 2.6% a notable improvement with respect to the previous MOOS-3 CRM. This paper provides minute details regarding both production and certification of the CRM, describing all aspect of method development, data analysis, uncertainty evaluation, value assignment, and providing a rich Electronic Supporting Material (ESM) inspired by the FAIR principles of data management [24], where the reader can find, review and reuse all raw data and software generated during this certification project.

## Material and methods

For utmost precision, sample and standard preparations were performed gravimetrically, allowing the nutrient content in MOOS-4 CRM to be reported as mass fraction. According to the IUPAC Gold Book, the symbol for denoting mass fraction is *w*. This convention was consistently adopted throughout the study. In the MOOS-4 certificate of analysis, the nutrient content is also reported in µmol/L [16].

### Reagents and materials

ACS grade reagents were obtained from MilliporeSigma (Oakville, ON, Canada): ammonium molybdate tetrahydrate (*w* = 99.98%), L-ascorbic acid (*w* ≥ 99%), N-(1-naphthyl) ethylenediamine dihydrochloride (*w* ≥ 98%), oxalic acid dehydrate (*w* ≥ 99.5%), potassium antimony(III) tartrate hydrate (*w* = 99.95%), potassium permanganate (*w* ≥ 99.0%), sodium chloride (*w* ≥ 99.95%), sodium hydroxide solution (10.0 M), sulfamic acid (*w* = 99.999%), sulfanilamide (*w* ≥ 99%), sulfuric acid concentrate (*w* = 95.5–96.5%), triethyloxonium tetrafluoroborate (*w* ≥ 97%). Concentrated hydrochloric acid (*w* = 36.5–38.0%) was obtained from ACP chemicals (Montreal, QC, Canada). Isotopically enriched nitrite (Na^15^NO_2_, *w*(^15^N) ≥ 98%) and nitrate (Na^15^NO_3_, *w*(^15^N) ≥ 98%) were obtained from Cambridge Isotope Laboratories (Tewksbury, MA, USA), whereas isotopically enriched silicate solution (*w*(^30^Si) = 550 mg/kg) was prepared by dissolving silicon-30-enriched single crystals in 25% *w*/*w* NaOH as reported previously [25]. High-purity water (exceeding ISO 3696 grade 1 standard) was generated in-house with a Thermo Scientific Gen-Pure UV xCAD plus system (18.2 MΩ cm at 25 °C). Low nutrient seawater was obtained from OSIL (Hampshire, UK).

Primary standards for nitrate (SRM 3185, lot 170309), phosphate (SRM 3186, lot 170606), and silicate (SRM 3150, lot 211509) were obtained from the National Institute of Standards and Technology (Gaithersburg, MD, USA), whereas primary standard for nitrite (67276, lot BCCK5026) was obtained from MilliporeSigma (Oakville, ON, Canada). For quality control, the NRC SALT-1 CRM was also analyzed [17, 26].

### Instrumentation

Mettler Toledo balances MS204S and XPE304 were calibrated against NRC reference masses (class F1) and used for gravimetric sample preparation and dilution of primary standards. A 25-mL volume pycnometer from MilliporeSigma was used for measuring seawater density. A Thermo Scientific Evolution 220 UV–visible spectrophotometer was used for measuring nitrite, phosphate and silicate in batch mode with 1- or 5-cm cuvettes (Hellma, Suprasil quartz). A 5973 Hewlett-Packard GC–MS system with a CTC CombiPAL autosampler was used for measuring nitrite and nitrate in negative chemical ionization (NCI) mode. Methane was used as NCI reagent gas at a set flow of 40% and the instrument was tuned accordingly to the standard autotune routine from manufacturer. Source and quadrupole temperatures were set at 150 °C. An Agilent 8800 ICP–MS interfaced with an Agilent 1200 series HPLC was used for measuring silicate. Details about instrumental settings can be found in the ESM.

### Analytical procedures

Three main methodologies were implemented for the determination of nitrite, nitrate, phosphate, and silicate in MOOS-4 CRM [27].

Headspace GC–MS was used for quantitation of nitrite and nitrate by isotope dilution. The method developed in this study was meant to improve analytical performance of our existing method [20, 21]. Briefly, the seawater sample was spiked with ^15^NO_2_^–^ and ^15^NO_3_^–^ and portioned in two aliquots. On the first aliquot, nitrite was reacted with Et_3_OBF_4_ to yield volatile EtONO. On the second aliquot, nitrite was eliminated with sulfamic acid and remaining nitrate was reacted with Et_3_OBF_4_ to yield EtONO_2_. In both cases, the derivatives were detected by static headspace GC–MS in negative ionization mode.

UV–vis spectrophotometry was used for quantitation of nitrite, phosphate and silicate by matrix-matching external calibration in batch mode. For each analyte, a portion of seawater was reacted to yield a colored derivative suitable for UV–vis detection. Traditional wet chemistries were used to convert phosphate into the phosphomolybdic complex [7, 8], silicate into a silicomolybdic complex [7], and nitrite into a diazo derivative [7]. The colored compounds were detected at 890 nm, 810 nm, and 541 nm respectively.

Finally, HPLC–ICP–MS was used for quantitation of silicate by isotope dilution [22]. Briefly, the seawater sample was spiked with a ^30^Si-enriched silicate solution. The analyte was separated from the saline matrix on a IonPac ICE-AS1 analytical column and detected by ICP–MS (O_2_ mode, *m*/*z* 28 and 30). Procedural details for all methods are reported in the ESM (Paragraphs S1 to S5).

## Results and discussion

### Preparation of the MOOS-4 CRM

The MOOS-4 is a costal seawater collected in February 2017 from Ketch Harbour, Nova Scotia, Canada, at a latitude of 44° 27′ 59.9″ N and longitude of 63° 33′ 31.3″ W. The seawater was sampled from a depth of about 10–12 m and its density was 1.0210 ± 0.0010 g/mL (22 ± 1 °C, *n* = 6, *k* = 2, pycnometry).

The sterility of the matrix was essential for the stability of the CRM. Initially, a simple ultrafiltration was explored as a mean to produce sterile seawater using hollow fiber tangential flow cartridges with a nominal molecular weight cutoff of 500 kDa (Cytiva model UFP-500-C-55). Unfortunately, the material was unstable and depletions of nitrate (−14%) and total nitrogen (−8%) were observed when the material was stored at room temperature for a period of three weeks. It is likely that such losses were caused by denitrifying bacteria that made it through the filtration process.

In order to eliminate the residual biological activity, the MOOS-4 was gamma irradiated with a minimum dose of 25.0 kGy. After irradiation, a portion of the material was left at room temperature and tested after 9 months. This time, no sign of nutrient depletion was observed.

Gamma irradiation induces inhomogeneity in nutrients composition [28]. Most notably, the process of irradiation is responsible for the formation of nitrite. Before radiation, nitrite was almost undetectable, whereas after irradiation a micromolar amount of nitrite could be measured. For this reason, the material was further reblended in a plastic carboy, bottled into its final 50 mL unit volume and kept refrigerated at 4–6 °C.

HDPE bottles used for MOOS-4 preparation were cleaned with 95% ethanol, rinsed three times with water, filled with ultrapure water and left soaking for a week. The same washing approach was implemented to clean the carboy used for reblending the material. Each MOOS-4 unit was tightly capped in order to ensure proper sealing and minimize evaporation losses in the long-term. When the MOOS-4 unit was kept at 40 °C for 63 days, a relative mass loss of −0.29% was observed, whereas under storage conditions at 4–6 °C the mass loss was only of −0.13% over a period of 6 years. For this test, the MOOS-4 units were not enclosed in sealed pouches.

### Determination of nitrite and nitrate by isotope dilution headspace GC–MS

Aqueous ethylation with Et_3_OBF_4_ allows conversion of nitrite and nitrate into derivatives which can be quantified with high-precision by isotope dilution GC–MS:

$${{\mathrm{NO}}_2}^{-}(\mathrm{aq})\;+\;{\mathrm{Et}}_3\mathrm O^{+}(\mathrm{aq})\;\rightarrow\;\mathrm{EtONO}\;+\;{\mathrm{Et}}_2\mathrm O$$

$${{\text{NO}}_{3}}^-(\text{aq})\;+\;{\text{Et}}_{3}{\text{O}}^{+}(\text{aq})\;\to\;{\text{EtONO}}_{2}\;+\;{\text{Et}}_{2}\text{O}$$

Although this single step aqueous derivatization chemistry is rapid and simple to implement [29, 30], the acid hydrolysis of this reagent—Et_3_O^+^(aq) + H_2_O → Et_2_O + EtOH + H^+^(aq)—is an obvious setback for the determination of nitrite and nitrate.

At low pH, nitrite and nitrate cannot be measured simultaneously because of the acidity of the medium which can catalyze partial conversion of nitrite into nitrate: NO_2_^−^(aq) + H^+^(aq) ⇄ HNO_2_ + ½ O_2_ → NO_3_^−^(aq) + H^+^(aq). Furthermore, acidity causes oxygen-exchange between NO_2_^−^(aq) and H_2_O, preventing the use of N^18^O_2_^−^ as internal standard.

In our previous study [20, 21], these issues were tackled by buffering the reaction medium with ammonium hydroxide (pH 10). This approach, however, has some practical limitations. First, the alkaline environment speeds up the hydrolysis of Et_3_O^+^(aq) reducing derivatization yield and method sensitivity. Second, the buffer contributes increasing the procedural blanks.

In this study, nitrite and nitrate were measured separately without pH adjustment. For the analysis of nitrite, the seawater sample was mixed with ^15^NO_2_^−^ and reacted with an aqueous solution of Et_3_OBF_4_. The nitrite loss due to acidity were accounted for by the internal standard. For the analysis of nitrate, the seawater sample was mixed with ^15^NO_3_^−^ and the residual nitrite was removed using sulfamic acid before derivatization with Et_3_OBF_4_.

EtONO and EtONO_2_ were detected by headspace GC–MS in NCI mode by monitoring *m*/*z* 31 and 32 for nitrite and *m*/*z* 46 and 47 for nitrate (see the mass spectra reported in Fig. S5).

**Fig. 1:**
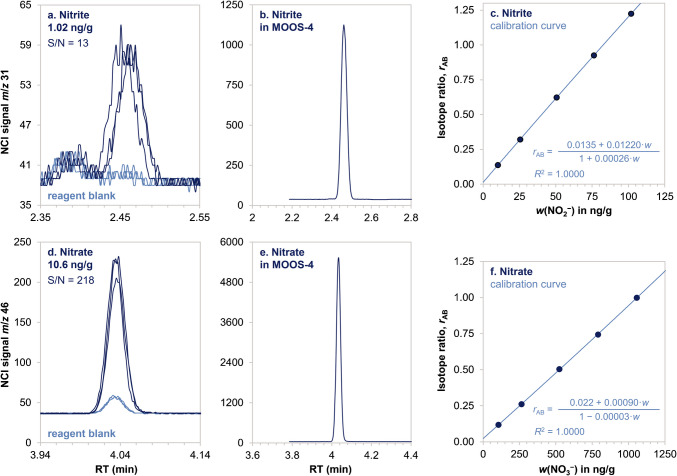
Determination of nitrite and nitrate by headspace GC–MS. **a** Overlaid chromatograms of three reagent blanks and three aqueous standards at 1.02 ng/g NO_2_^−^. **b** Nitrite signal in MOOS-4. **c** Calibration curve in the 10–100 ng/g NO_2_^−^ range. **d** Overlaid chromatograms of three reagent blanks and three aqueous standards at 10.6 ng/g NO_3_^−^. **e** Nitrate signal in MOOS-4. **f** Calibration curve in the 100–1000 ng/g NO_3_^−^ range

The use of isotopically enriched internal standards was beneficial to account for analyte losses during sample preparation and for signal suppression due to the high chloride matrix. Although signal suppression was 22% for nitrite and 50% for nitrate, the method was still very sensitive. As reported in Fig. 1, the method could achieve detection limits of 0.29 ng/g (6.4 nmol/L) and 0.30 ng/g (4.8 nmol/L) for nitrite and nitrate using only 2 mL of seawater. Such figures of merit are promising for potential applications to oligotrophic seawaters where the nutrient levels are in the nanomolar range [14]. In Fig. 1, a 5-point calibration curve is reported for nitrite (10–100 ng/g) and nitrate (0.11–1.1 µg/g). These ranges can easily be extended in both directions depending on the analytical requirements. The method is also very selective as the analytes are first separated from the matrix under the form of volatile derivatives and then separated again by gas chromatography, providing very clean signals (Fig. 1).

High-precision quantitation was obtained using isotope dilution. A multi-level calibration curve was fitted with a rational function—*y* = (*a*_0_ + *a*_1_·*x*)/(1 + *a*_2_·*x*) [23]—and the uncertainty was evaluated using error propagation [31]. Such calculations were automated using a custom Excel function named ResultIDMS_GS which can be found in Module 5 of the Excel file reported in ESM (see also Paragraph S1). In MOOS-4, nitrate and nitrite were measured with a relative standard uncertainty of 1.8% and 0.4%, respectively. The larger uncertainty on nitrate was due to the difficulty controlling laboratory contaminations, hence nitrate measurement precision could potentially be improved.

**Fig. 2:**
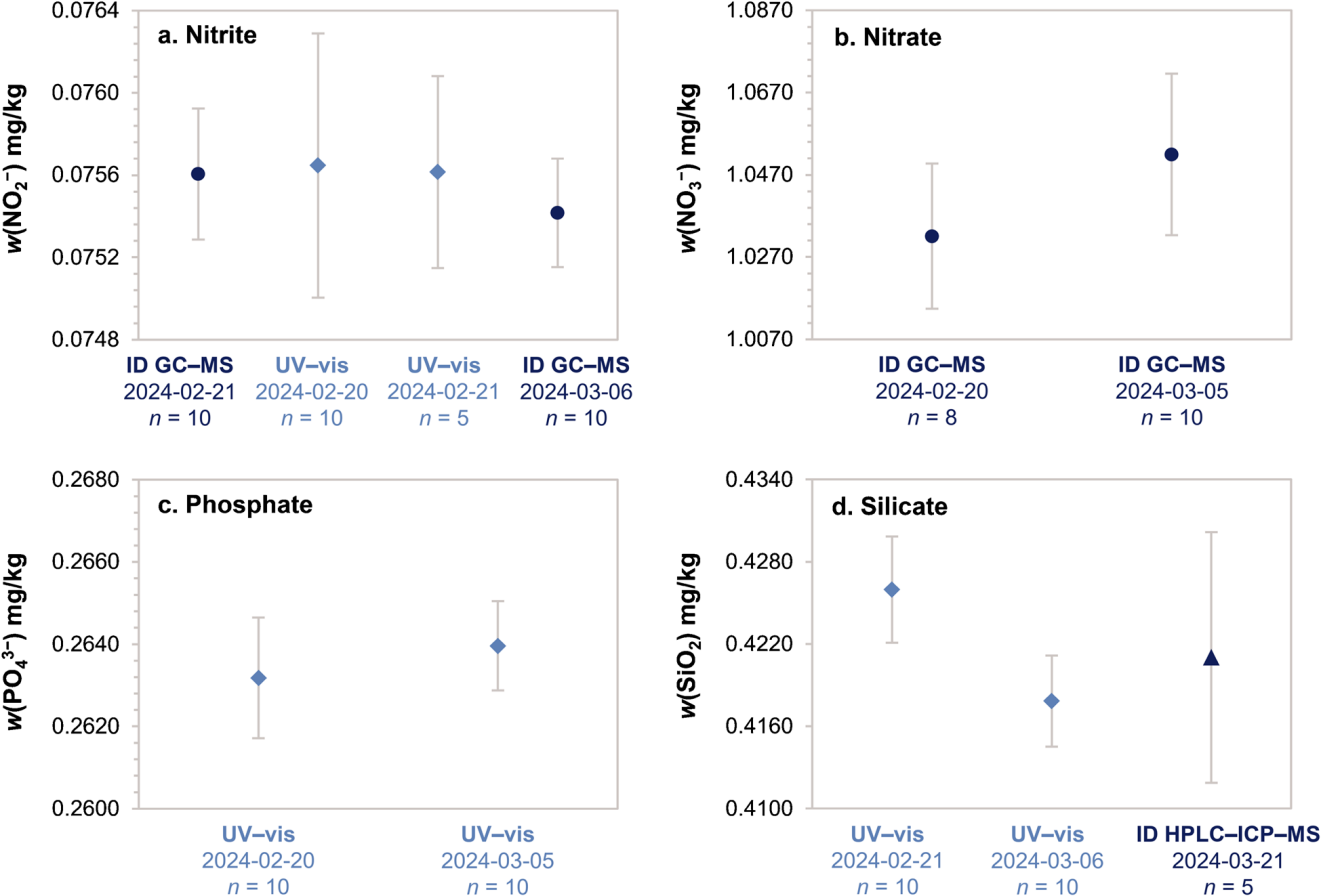
Determination of nutrients in MOOS-4 using different methods in different days. Isotope dilution results are reported in dark blue (circles for ID GC–MS and triangles for ID HPLC–ICP–MS). Matrix-matching external calibration results are reported in light blue diamonds (UV–vis). Dates and number of measurements (*n*) are reported for each value

The accuracy of this approach for nitrate measurement was corroborated during the CCQM K161 laboratory intercomparison, where the data generated applying this method was in perfect agreement with the KCRV reference value (degree of equivalence = −0.002 ± 0.074 mg/kg NO_3_^−^, *k* = 2) [32]. The accuracy of nitrite data was demonstrated by the tight agreement with an independent technique based on UV–vis spectrophotometry (Fig. 2)

### Determination of nitrite, phosphate, and silicate by spectrophotometry with matrix-matching calibration

Over the years, the NRC reference procedures for the spectrophotometric determination of nitrite, phosphate, and silicate in seawater have been under constant development with the aim to perfection their performance [17]. Further validation figures are presented in this study.

Spectrophotometric methods are based on traditional wet chemistries for color development which have been in use for many years (for technical details see Paragraphs S2–S4) [7]. Briefly, the sample was transferred into plastic vial and its mass (*m*_S_) was recorded. Then, color development was obtained in batch mode by adding the required reagents. The mass of sample plus reagents (*m*_F_) was also recorded. The reaction was kept at room temperature for a certain time before the absorbance of the solution was read. Five calibration standards were prepared in low nutrient seawater (OSIL) and were processed in the same way. For utmost precision, the nutrient composition of the calibration standard in the middle of the curve was matched with that of the MOOS-4. As illustrated in the ESM Excel file, the absorbance readings were corrected for five effects which could have biased the quantitation. First, the signals were corrected for the residual absorbance of the underivatized matrix. This operation was required for normalizing the responses to the same background level and was inspired by EPA 366 [33]. Second, the signals of the calibration standards were corrected for the blank contribution carried by the low nutrient seawater used for the preparation of the standards. The blank levels for nitrite and phosphate were negligible whereas for silicate, the standard in the middle of the calibration curve required a 10% blank correction. Third, in order to correct for the small mismatch in the gravimetric composition of samples and standards, the signals were multiplied by the *m*_F_/*m*_S_ factor [34]. Fourth, in order to correct for the slight density differences between samples and standards, all signals were divided by the densities of the corresponding media. This correction was required because the calibration curve was obtained plotting signals against the mass fraction of the analytes. Since the absorbance is proportional to concentration, samples or standards with the same mass fraction but with different density (hence different concentration) would yield different signals. Although minimal, this density correction allowed the use of primary standard prepared gravimetrically. Fifth, the signals were corrected for any matrix effects due to signal suppression/enhancement. The preparation of the external calibration in a matrix-matched environment was aimed to minimize such effects, however some degree of mismatching between the matrices of samples and standards should be taken into account for high-precision work. In order to quantify the matrix effects, the ratio (α) between the slope of the calibration curve prepared within the sample matrix and the slope of the calibration curve obtain in low nutrient seawater was measured. For phosphate and silicate, α did not differ from 1; hence, no matrix effects were detected for these two analytes. For nitrite, as reported in Fig. S6, a small but significant matrix effect was observed with α(NO_2_^−^) = 1.0096 (*u* = 0.0014, *u*_R_ = 0.14%). To take into account this contribution, the nitrite signals for MOOS-4 were divided by α(NO_2_^−^).

At this point the calibration curves were obtained by plotting the (corrected) absorbance against the mass fraction of the primary standards. Both linear (*y* = *a*_0_ + *a*_1_·*x*) and quadratic (*y* = *a*_0_ + *a*_1_·*x + a*_2_·*x*^2^) models were used for quantitation. Since the difference between the two models was negligible, the results arising from the simpler linear model were used for value assignment. Error propagation was implemented to estimate the uncertainty on the single measurement [31]. Such calculations were automated using a custom Excel function named ResultEXLN_GS which can be found in Module 4 of the Excel file reported in ESM.

The accuracy of this approach was demonstrated during the CCQM K161 laboratory intercomparison, where phosphate data generated applying this method was in agreement with the KCRV reference value (degree of equivalence = 0.938 ± 5.638 µg/kg PO_4_^3−^ as P, *k* = 2) [32]. Furthermore, the accuracy for nitrite and silicate was demonstrated by the agreements with independent isotope dilution techniques as reported in Fig. 2.

Two additional validation experiments were performed. First, possible interference of silicate on the signal of phosphate was studied in the 0.0–2.8 mg/kg SiO_2_ range. As reported in Fig. S7, no interference was observed. Second, the effect of temperature on the kinetics of color development was studied at 4 °C (typical storage conditions for seawater samples) and 20 °C (typical temperature for seawater testing). A standard solution matched to the nutrient composition found in MOOS-4 was prepared in low nutrient seawater and spit into six aliquots. Three aliquots were kept at 4 °C, while the other were kept at 20 °C for 24 h. These solutions were then tested for nutrients using the spectrophotometric methods described in Paragraphs S2–S4. In the case of nitrite and phosphate, no difference was found between the samples stored at 4 and 20 °C. For silicate, the temperature of the sample had a remarkable effect. The average signal of the samples at 20 °C was 0.14187 (*u* = 0.00073, *u*_R_ = 0.5%, *n* = 3) whereas the average signal of the sample at 4 °C was 0.1089 (*u* = 0.0050, *u*_R_ = 4.6%). Therefore, for silicate quantitation, the samples were equilibrated at room temperature before starting the analysis.

### Determination of silicate by isotope dilution HPLC–ICP–MS

Direct determination of silicate in seawater was performed by HPLC–ICP–MS without the need for analyte derivatization. In this project, previous procedure [22] was improved and implemented on an Agilent 8800 triple quadrupole ICP–MS. An equal volume of sample (or primary standard) and of internal standard where mixed and injected in the HPLC system. An isotopically enriched aqueous solution of ^30^Si-silicate was used as the internal standard. Although the ion exclusion AS1 Dionex column could efficiently separate silicate from the saline matrix (i.e., no matrix suppression was observed for the silicate signal), the use of the internal standard was essential to compensate for drifts during chromatography. The elution was performed with aqueous HCl, sufficiently diluted to avoid damages to the HPLC system, but enough for elution of silicate.

Due to the high concentration of silicate in MOOS-4, acquisition was performed in O_2_ mode to reduce the number of counts on *m*/*z* 28 and 30. The gas phase reaction in the collision cell was aimed to reduce the amount of Si^+^ by promoting transition into SiO^+^. Even with this intentional signal reduction, the pulse counting detector became saturated during the analysis of MOOS-4, triggering the switch between pulse and analog detector in the middle of silicate elution. This discontinuity was responsible for poor chromatographic peak shapes. To overcome the issue, ICP–MS was forced to acquire data only in analog mode.

**Fig. 3:**
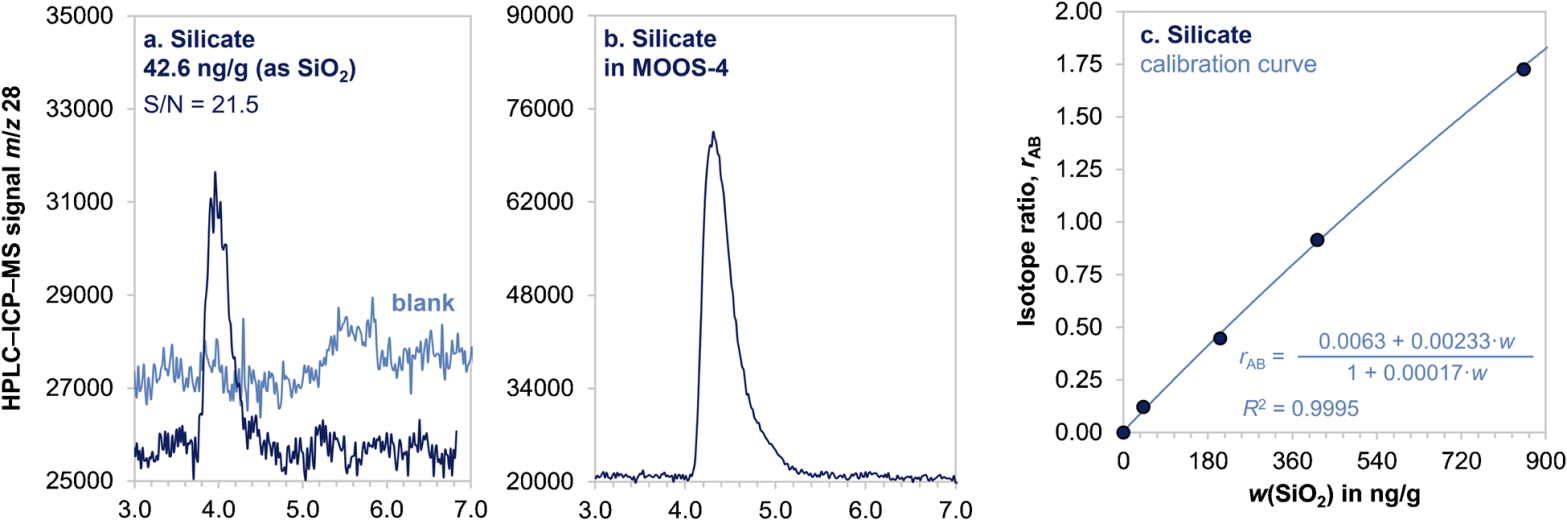
Determination of silicate by HPLC–ICP–MS. **a** Overlaid chromatograms of a reagent blank and an aqueous standard at 42.6 ng/g SiO_2_. **b** Silicate signal in MOOS-4. **c** Calibration curve in the 40–850 ng/g SiO_2_ range

High-precision quantitation of silicate was obtained by isotope dilution with a multi-level calibration curve as described in the “Determination of nitrite and nitrate by isotope dilution headspace GC–MS” section. The relative standard uncertainty for the mass fraction of silicate in MOOS-4 was 1.7% with a LOQ of 20 ng/g as SiO_2_ (Fig. 3).

The procedural blanks were not of concern for quantitation of silicate in MOOS-4. Nevertheless, it was observed that some silicate leached from the polypropylene autosampler vials after 24 h storage. To avoid contamination, the samples were run within the same day of their preparation. As reported in Fig. 2, the result generated using HPLC–ICP–MS were in perfect agreement with those generated with the classical UV–vis method based on silicon-molybdenum derivatization chemistry.

### Metrological traceability

For the determination of nitrate, phosphate, and silicate, traceability to the SI system was established through the NIST SRM 3185, 3186, and 3150 respectively. These standards were gravimetrically diluted and used for calibration.

For the determination of nitrite, a standard from MilliporeSigma was employed (p/n 67276, Lot BCCK5026). Such solution was certified for *w*(NO_2_^−^) = 1001 ± 4 mg/kg (*k* = 2). As described in Paragraph S6, the mass fraction of this standard was further verified by isotope dilution against NIST SRM 3185. In practice, an aliquot of the nitrite solution was reacted with permanganate in acid medium: 2MnO_4_^−^ + 5 NO_2_^−^ + 6H^+^ → 2Mn^2+^ + 5 NO_3_^−^ + 3H_2_O. The reaction conditions for this oxidation were inspired by a traditional titration method [35].

The resulting nitrate was quantified by headspace IDMS GC–MS with the same methodological approach employed for seawater (“Determination of nitrite and nitrate by isotope dilution headspace GC–MS” section). The measurement was repeated three times and yield *w*(NO_2_^−^) = 1004.1 ± 5.8 mg/kg (*k* = 2) which is in excellent agreement with the value reported by MilliporeSigma.

In order to further validate this procedure, it was demonstrated that the oxidation conditions employed for nitrite conversion to nitrate were quantitative (no nitrite was detected after the oxidation), and no detectable levels of nitrate were found in the original nitrite standard.

This experiment demonstrated that the method proposed for the measurement of nitrate by isotope dilution can be adapted for quantitation of nitrite ions against a nitrate primary standard. This approach can be valuable for direct purity assessment of nitrite standards.

### Certification of MOOS-4: stability

Previous seawater batch used for the preparation of MOOS-1, MOOS-2, and MOOS-3 was suitable for over two decades, demonstrating high chemical stability of nutrients within seawater matrix [28]. For example, MOOS-3 was made in 2013 starting from a seawater batch collected in 1996 and was monitored for 8 years up to 2021 without detecting stability issues (Fig. S8).

From a stability perspective, biological activity is the most critical vulnerability for seawater nutrients CRMs. Biological activity can result in severe alteration of nutrients composition, hence stability of nutrients CRMs can only be ensured sterilizing the material. The MOOS-4 was sterilized by gamma radiation using the same approach employed for previous CRMs [28].

In this study, the stability of MOOS-4 was assessed in an accelerated study using the high-precision methods described previously. Two sets of four MOOS-4 units were incubated at 21 °C and 40 °C, respectively. Two additional MOOS-4 units were kept at 4 °C (storage condition) for reference. After 12 days, one unit kept at 21 °C and one unit kept at 40 °C were moved into in the fridge (4 °C reference condition). This operation was repeated at the 27th, 35th, and 43rd day after initial incubation. Thereafter, all ten units were kept at 4 °C for 8 months before analysis. As reported in ESM (Excel File, Tab: SHORT_HOM), the eight accelerated units and the two reference ones were analyzed simultaneously in randomized order. Each unit was analyzed two times and its response was normalized to the reference. In all cases, no major variations in the nutrients’ response were detected, proving a successful sterilization and preparation process. As shown in Fig. S9, no trends were observed for nitrite, nitrate, and silicate, therefore zero uncertainty was assigned to stability (Table 1).

**Table 1:** MOOS-4 certified property values and uncertainty components

Analyte	Form	*w*	*U*(*w*)	*u*_R,SHORT_	*u*_R,LONG_	*u*_R,HOM_	*u*_R,CHAR_	*u*_R,PRIM_
Nitrite	NO_2_^−^	0.07552	0.00056	0%	0%	0%	0.24%	0.29%
Nitrate	NO_3_^−^	1.041	0.027	0%	0%	0.20%	1.3%	0.092%
Phosphate	PO_4_^3−^	0.2637	0.0029	0.06%	0.39%	0%	0.33%	0.18%
Silicate	as SiO_2_	0.4214	0.0076	0%	0%	0%	0.89%	0.12%

*w*, mass fraction in mg/kg; *U*(*w*), expanded uncertainty on mass fraction in mg/kg (*k* = 2); *u*_*R,SHORT*_, relative standard uncertainty for stability under transportation conditions; *u*_*R,LONG*_, relative standard uncertainty for stability under storage conditions; *u*_*R,HOM*_, relative standard uncertainty due to within units homogeneity; *u*_*R,CHAR*_, relative standard uncertainty due to characterization; *u*_*R,PRIM*_, relative standard uncertainty due to the uncertainty on the mass fraction of the primary standard

**Fig. 4:**
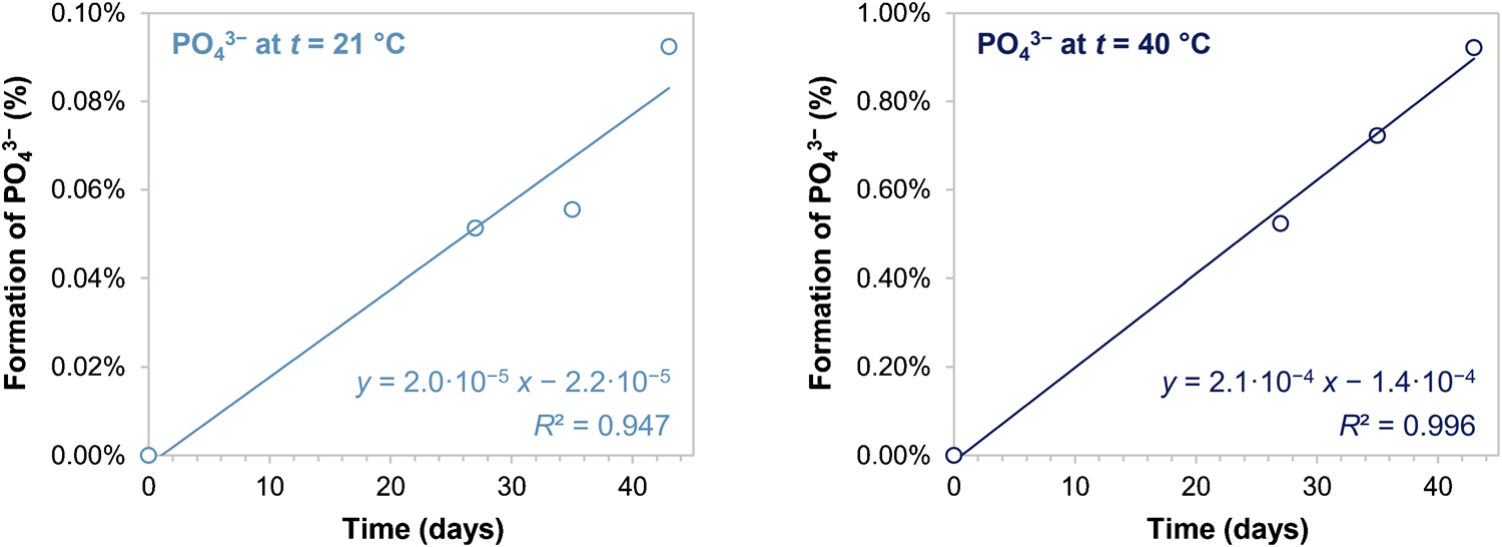
Accelerated stability study for phosphate in MOOS-4. Minor phosphate formation was observed when the material was kept at 21 °C (~0.1% in 40 days) and at 40 °C (~ 0.9% in 40 days). Formation of phosphate is reported in percentage with respect to the endogenous content of phosphate

Conversely, phosphate formation was observed when the material was kept at elevated temperature. Formation of phosphate seems to be a zero order reaction with an estimated Q_10_ temperature coefficient of 3.5 (Fig. 4). This phosphate formation was likely due to the hydrolysis of phosphorous-containing compounds which would not otherwise partake into the molybdenum blue reaction [36, 37]. This effect was overall very minor as the mass fraction of phosphate increased only by 0.9% after 43 days at 40 °C. Nevertheless, this effect was kept in consideration for the estimation of uncertainty due to stability. For the relative standard uncertainty due to stability under transportation conditions (*u*_R,SHORT_) a 10 days lag at 30 °C was considered the worst-case scenario whereas a 4 years period at 5 °C was used for the relative standard uncertainty component due to stability under storage conditions (*u*_R,LONG_). A Q_10_ of 3.5 allowed to estimate a *u*_R,SHORT_ of 0.06% and a *u*_R,LONG_ of 0.39%.

As a risk mitigation strategy for long-term stability, the MOOS-4 will be monitored every 2 years throughout the lifecycle of the CRM, which will likely be longer than 4 years.

### Certification of MOOS-4: homogeneity

Since the analytes are ionic compounds at the part-per-million level and are fully soluble in water, there is no reason to question the within bottle homogeneity, however the within unit homogeneity was evaluated in order to demonstrate suitability of the preparation process. Nutrients’ measurement methods required minimum sample volume of 6.0 mL for phosphate, 3 mL for silicate, and 2 mL for both nitrite and nitrate.

Nitrite, nitrate, and silicate were stable under the condition tested in the accelerated study described in the previous section; therefore, the stability data were also used to assign the within unit homogeneity. As reported in ESM, a one-way ANOVA was applied to the nutrients data from ten units analyzed in duplicate [38]. Only for nitrate a minor homogeneity component was found (Table 1), whereas for the other nutrients no heterogeneity could be detected. The uncertainty of the method used for this test was considered for the estimation of the uncertainty due to characterization, as reported in the following paragraph. Hence, *u*_R,HOM_ was not further inflated to account for method contribution (Table 1) [39].

For phosphate, the homogeneity was estimated using the DerSimonian and Laird method (DSL) [40] on the data generated for value assignment. DSL calculation was performed using the R programming language (DSL function under the metRology package [41]). As reported in ESM, no uncertainty component was found for phosphate (τ = 0).

### MOOS-4 characterization and value assignment

Ten units of MOOS-4 were sampled across the batch and used for characterization. The measurements were carried out using multiple methods over several days (Fig. 2, raw data can be found in ESM). For increasing the independency between the data generated at different times, each day a new set of calibration standards was prepared from direct dilution of the primary standard stock solutions. SALT-1 CRM was run within each sequence for quality control of nitrate, phosphate, and silicate [17]

Linear pooling was used to combine data generated within the same day using the same analytical method and the results are shown in Fig. 2. Linear pooling calculation was performed using the NIST consensus builder [42]. The data in Fig. 2 were further reduced using the DerSimonian-Laird method. The μ value returned by DSL was used for value assignment (Table 1). For nitrite, nitrate, and phosphate, the τ value from DSL was zero; therefore, the uncertainty component due to characterization (i.e., *u*_R,CHAR_, Table 1) was simply the standard error returned by DSL. For silicate, DSL returned the following values: μ = 0.4214 mg/kg, *s* = 0.0029 mg/kg, τ = 0.0024 mg/kg. In this case, the uncertainty component due to characterization was obtained combining *s* and τ: *u*_R,CHAR_ (SiO_2_) = (*s*^2^ + τ^2^)^1/2^/μ = 0.89%.

The combined standard uncertainty was obtained by combining the uncertainty components associated to stability, homogeneity, characterization and primary standard: *u*_R_ = (*u*^*2*^_R,SHORT_ + *u*^2^_R,LONG_ + *u*^2^_R,HOM_ + *u*^2^_R,CHAR_ + *u*^2^_R,PRIM_)^1/2^. The expanded uncertainty (*U*_R_ = *k*·*u*_R_) was obtained on a coverage factor *k* = 2. All calculation for characterization and value assignment can be found in ESM (Excel File, Tab: CHAR_HOM) and reference to the certificate of analysis can be found here [16].

## Conclusion

In this study, production and certification of the MOOS-4 certified reference material for nutrients in seawater were described with particular attention to the analytical methods developed and optimized for high-precision quantitation. The proposed analytical work is important for the characterization of CRMs and serves as a benchmark to cross-validate the performance of high-throughput methodologies used by oceanographers, advancing towards the very tight uncertainty levels required for environmental studies.

Nitrite, nitrate, phosphate, and silicate could be measured within a relative expanded uncertainty of 0.8%, 2.6%, 1.1%, and 1.8% (*k* = 2). With respect to the previous version of this material, the uncertainty on phosphate and nitrite were improved by a factor of eight and two, respectively.

Nitrite, nitrate, and silicate could be measured with advanced primary isotope dilution methodologies which, in the case of nitrite and silicate, returned values in excellent agreement with the traditional spectrophotometric methods. Quantitation was obtained using a multiple-point calibration curve which was fit with a rational function. This approach was mathematically equivalent with respect to traditional isotope dilution equations, but simpler to implement. The only analyte which still required the development of a dedicated isotope dilution method is phosphate which was measured using the molybdenum blue approach.

Although only one method was used for quantitation of nitrate and phosphate, the validity of these procedures was recently demonstrated in a successful intercomparison study (CCQM-K161). As a proposition for the future, we commit to develop a higher-order isotope dilution method also for phosphate and to expand the production of NRC nutrients seawater CRMs to embrace a wider range of concentrations and salinities.

## Supplementary Information

Below is the link to the electronic supplementary material.

Supplementary file1 (PDF 1495 KB)

Supplementary file1 (XLSM 887 KB)

## Data Availability

All data generated or analyzed during this study are included in this published article and its supplementary information files.
