# Supplementary material for: High-precision determination of nitrite, nitrate, phosphate, and silicate for the characterization of MOOS-4 certified reference material for nutrients in seawater
Source: Anal Bioanal Chem. 2025 May 31;417(18):4111–21. doi: 10.1007/s00216-025-05928-7 (PMC12276123; doi:10.1007/s00216-025-05928-7)
Supplement: Supplementary file 1 — Supplementary file1 (PDF 1495 KB) [file 216_2025_5928_MOESM1_ESM.pdf]

**Analytical and bioanalytical chemistry**

*Electronic supplementary material*

# High-precision determination of nitrite, nitrate, phosphate and silicate for the characterization of MOOS-4 Certified Reference Material for nutrients in seawater

Enea Pagliano, Zuzana Gajdosechova

*Metrology Research Center, National Research Council Canada, 1200 Montreal Road, K1A 0R6, Ottawa, Ontario, Canada*

Email: enea.pagliano@nrc-cnrc.gc.ca

## Table of Content

**Paragraph S1.** Headspace GC–MS determination of nitrite and nitrate in seawater...... 2
**Paragraph S2.** Spectrophotometric determination of nitrite in seawater........................ 9
**Paragraph S3.** Spectrophotometric determination of phosphate in seawater.............. 12
**Paragraph S4.** Spectrophotometric determination of silicate in seawater ................... 15
**Paragraph S5.** HPLC–ICP–MS determination of silicate in seawater ......................... 18
**Paragraph S6.** Headspace GC–MS standardization of nitrite (67276 MilliporeSigma). 20
**Figure S1** Manifold for the purification of triethyloxonium tetrafluoroborate................. 26
**Figure S2** Purification of triethyloxonium tetrafluoroborate.......................................... 27
**Figure S3** GC–MS chromatograms of nitrite in MOOS-4............................................ 28
**Figure S4** GC–MS chromatograms of nitrate in MOOS-4........................................... 29
**Figure S5** NCI mass spectra of nitrite and nitrate ethyl-derivatives ............................. 30
**Figure S6** Determination of nitrite by spectrophotometry: rotational matrix effect ........ 31
**Figure S7** Silicate interference on phosphate signal at 890 nm.................................. 32
**Figure S8** Long-term stability study on MOOS-3 ........................................................ 33
**Figure S9** Accelerated stability study for nitrate, nitrite and silicate in MOOS-4 .......... 34
**References** ................................................................................................................ 35

**Paragraph S1.** Headspace GC–MS determination of nitrite and nitrate in seawater

1. **Preparation of the internal standard solution**

   *Raw data and calculations:*
   ESM Excel file, Tab: RND, Line 666

   *Composition:*
   $w(^{15}NO_3^-)$ ~ 10.66 µg/g, $w(^{15}NO_2^-)$ ~ 0.810 µg/g

   *Medium:*
   Ultrapure water adjusted at pH 10-11 (which can be obtained by adding 50 µL of NaOH 10 M to 500 mL of ultrapure water)

2. **Preparation of the primary standard solutions**

   *Raw data and calculations:*
   ESM Excel file, Tab: CHAR_HOM (for examples see Lines 322-325 and 812-815)

   *Composition:*

| | Target $w(NO_2^-)$ µg/g | Target $w(NO_3^-)$ µg/g |
|---|---|---|
| Standard 1 | 0.0388 | 0.531 |
| Standard 2 | 0.0776 | 1.06 |
| Standard 3 | 0.155 | 2.12 |
| Standard 4 | 0.233 | 3.19 |

   *Medium:*
   Ultrapure water

3. **Purification of triethyloxonium tetrafluoroborate**

   *Preparation:*
   1.0 g of $Et_3OBF_4$ was transferred into a PFA vial allowing argon flushing as simplified in Fig. S1. The vial headspace was flushed with argon for 5 min at room temperature. Then the PFA vial was transferred in a hot block (50 °C) and flushed with argon for 30 min. The PFA vial was removed from the hot block and flushed with argon for another 10 min. The PFA vial was closed with a solid cap and kept at −20 °C. $Et_3OBF_4$ purification is required for reducing nitrate contamination. This step can usually be omitted when $Et_3OBF_4$ is used for nitrite analysis.

   *Safety precaution:*
   $Et_3OBF_4$ is a strong alkylating agent which need to be handled accordingly to its SDS. All manipulations of the reagent should be performed under a fume hood wearing suitable PPE. $Et_3OBF_4$ is stored at −20 °C protected from light.

4. **Preparation of the blends for isotope dilution quantitation**

*Raw data and calculations:*
ESM Excel file, Tab: CHAR_HOM, Lines 328-354

*Preparation:*
5.0 mL of sample (or primary standard solution) was transferred into a plastic bottle along with 0.5 mL of internal standard solution. The masses of the solutions were recorded. The blends were thoroughly mixed.

5. **Preparation of the blends for blank correction**

*Raw data and calculations:*
ESM Excel file, Tab: CHAR_HOM, Lines 328-354

*Preparation:*
5.0 mL of blank medium was transferred into a plastic vial along with 0.5 mL of internal standard solution and thoroughly mixed. Blank media without internal standard were prepared.

*Blank media:*
Ultrapure water and 3.5 % NaCl in ultrapure water.

6. **Derivatization chemistry for the determination of nitrite**

*Preparation:*
2.0 mL of the isotope dilution blends (prepared at points 4 and 5) were transferred into a 10 mL headspace vials suitable for CTC CombiPAL autosampler. An aliquot of 0.04 mL of triethyloxonium tetrafluoroborate aqueous solution was added (see point 9) and mixed. After 30 min, the resulting EtONO derivative was measured by headspace GC–MS.

7. **Derivatization chemistry for the determination of nitrate**

*Preparation:*
2.0 mL of the isotope dilution blends (prepared at points 4 and 5) were transferred into a 10 mL headspace vials suitable for CTC CombiPAL autosampler. An aliquot of 0.1 mL of 1 % sulfamic acid aqueous solution was added (see point 8) and mixed. At this point 0.04 mL of triethyloxonium tetrafluoroborate aqueous solution was added (see point 9) and mixed. After 30 min, the resulting $EtONO_2$ derivative was measured by headspace GC–MS.

8. **1 % sulfamic acid aqueous solution**

*Preparation:*
0.3 g of sulfamic acid was dissolved in 29.7 g of ultrapure water. The solution was kept in a plastic bottle at 4 °C for no longer than 1 month.

## 9. Triethyloxonium tetrafluoroborate aqueous solution

*Preparation:*

The purified 1.0 g $Et_3OBF_4$ was removed from the freezer (see point 3). An aliquot of 1.0 mL of ultrapure water previously cooled to 4 °C was added. The $Et_3OBF_4$ was quickly dissolved and used within 5 min. Since $Et_3OBF_4$ is unstable in water, leftovers of this solution were discarded.

*Safety precaution:*

$Et_3OBF_4$ is a strong alkylating agent which need to be handled accordingly to its SDS. All manipulations of the reagent should be performed under a fume hood wearing suitable PPE. $Et_3OBF_4$ is stored at −20 °C protected from light.

## 10. Headspace GC–MS method for detection of nitrite (EtONO)

*Instrumental conditions for CTC headspace incubation and sampling:*

| | |
|---|---|
| Injection volume: | 750 µL |
| Syringe size: | 2.5 mL for headspace |
| Incubation temperature: | 60 °C |
| Incubation time: | 2 min |
| Syringe temperature: | 70 °C |
| Agitator speed: | 500 rpm |
| Fill speed: | 100 µl/s |
| Fill strokes: | 3 |
| Pullup delay: | 1000 ms |
| Injection speed: | 500 µl/s |
| Pre inject delay: | 500 ms |
| Post inject delay: | 500 ms |
| Flush time: | 5 min |
| GC runtime: | 12 min |

*Instrumental conditions for GC separation:*

*OVEN*

| | |
|---|---|
| Equilibration time: | 0.5 min |
| Initial temp: | 30 °C |
| Isotherm time: | 2.5 min |
| Ramp 1: | 20 °C/min up to 140 °C |
| isotherm time: | 0.0 min |
| Run time: | 8.00 min |

*FRONT INLET (SPLIT/SPLITLESS)*

| | |
|---|---|
| Mode: | Split |
| Initial temp: | 120 °C |
| Liner type: | 1 mm strait |
| Split ratio: | 7:1 |
| Split flow: | 6.9 mL/min |
| Total flow: | 11.2 mL/min |
| Gas saver: | On |

| | |
|---|---|
| Saver flow: | 20.0 mL/min |
| Saver time: | 2.00 min |
| Gas type: | Helium |
| *COLUMN* | |
| Capillary column: | DB-624 (6%-cyanopropyl-phenyl-94%-dimethyl polysiloxane) |
| Model number: | J&W 122-1334 |
| Nominal length: | 30 m |
| Nominal diameter: | 250.00 µm |
| Nominal film thickness: | 1.40 µm |
| Mode: | constant flow |
| Initial flow: | 1.0 mL/min |
| MSD transfer line temp: | 220 °C |

*Instrumental conditions for MS detection:*

| | |
|---|---|
| Solvent delay: | 2.1 min |
| EMV mode: | Gain Factor |
| Gain factor: | 1.0 |
| Resulting EM: | 1118 V |
| SIM parameters ion 1: | *m*/*z* 31 (dwell time 50 ms) |
| SIM parameters ion 2: | *m*/*z* 32 (dwell time 50 ms) |
| MS source temp: | 150 °C |
| MS quad temp: | 150 °C |
| Timed events: | MS OFF at 3.0 min |

*Retention time and raw data:*

Under this program, nitrite (under the form of EtONO) elutes at 2.46 min (Fig S3). The integration of the signals was obtained using the Agilent MassHunter software (B.06.00, Built 6.0.633.0, Agile integrator, 2012). Areas at *m*/*z* 31 and *m*/*z* 32 were exported in ESM Excel file, Tab: RAW_DATA, Lines 389 and 735.

## 11. Headspace GC–MS method for detection of nitrate ($EtONO_2$)

*Instrumental conditions for CTC headspace incubation and sampling:*

| | |
|---|---|
| Injection volume: | 750 µL |
| Syringe size: | 2.5 mL for headspace |
| Incubation temperature: | 60 °C |
| Incubation time: | 2 min |
| Syringe temperature: | 70 °C |
| Agitator speed: | 500 rpm |
| Fill speed: | 100 µl/s |
| Fill strokes: | 3 |
| Pullup delay: | 1000 ms |
| Injection speed: | 500 µl/s |
| Pre inject delay: | 500 ms |
| Post inject delay: | 500 ms |

| | |
|---|---|
| Flush time: | 5 min |
| GC runtime: | 7 min |

*Instrumental conditions for GC separation:*

| | |
|---|---|
| *OVEN* | |
| Equilibration time: | 0.5 min |
| Initial temp: | 50 °C |
| Isotherm time: | 1.5 min |
| Ramp 1: | 20 °C/min up to 140 °C |
| Isotherm time: | 0.0 min |
| Run time: | 6.00 min |
| *FRONT INLET (SPLIT/SPLITLESS)* | |
| Mode: | Split |
| Initial temp: | 120 °C |
| Liner type: | 1 mm strait |
| Split ratio: | 7:1 |
| Split flow: | 6.9 mL/min |
| Total flow: | 11.2 mL/min |
| Gas saver: | On |
| Saver flow: | 20.0 mL/min |
| Saver time: | 2.00 min |
| Gas type: | Helium |
| *COLUMN* | |
| Capillary column: | DB-624 (6%-cyanopropyl-phenyl-94%-dimethyl polysiloxane) |
| Model number: | J&W 122-1334 |
| Nominal length: | 30 m |
| Nominal diameter: | 250.00 µm |
| Nominal film thickness: | 1.40 µm |
| Mode: | constant flow |
| Initial flow: | 1.0 mL/min |
| MSD transfer line temp: | 220 °C |

*Instrumental conditions for MS detection:*

| | |
|---|---|
| Solvent delay: | 3.7 min |
| EMV mode: | Gain Factor |
| Gain factor: | 1.0 |
| Resulting EM: | 1118 V |
| SIM parameters ion 1: | *m*/*z* 46 (dwell time 50 ms) |
| SIM parameters ion 2: | *m*/*z* 47 (dwell time 50 ms) |
| MS source temp: | 150 °C |
| MS quad temp: | 150 °C |
| Timed events: | MS OFF at 5.2 min |

*Retention time and raw data:*

Under this program, nitrate (under the form of $EtONO_2$) elutes at 4.03 min (Fig S4). The integration of the signals was obtained using the Agilent MassHunter software (B.06.00, Built 6.0.633.0, Agile integrator, 2012). Areas at *m*/*z* 46 and *m*/*z* 47 were exported in ESM Excel file, Tab: RAW_DATA, Lines 283 and 586.

## 12. Calculation of isotope ratio and blank correction

*Raw data and calculations:*

*Nitrite*: find the example in ESM Excel file, Tab: RAW_DATA, Line 389.
The isotope ratio $r_{AB}$ for nitrite is the ratio between the chromatographic area at *m*/*z* 31 and at *m*/*z* 32. Standard uncertainty $u(r_{AB})$ was estimate using repeatability standard deviation. Procedural blank for nitrite is not usually significant.

*Raw data and calculations:*

*Nitrate*: find the example in ESM Excel file, Tab: RAW_DATA, Line 283.
The isotope ratio $r_{AB}$ for nitrate is the ratio between the chromatographic area at *m*/*z* 46 and at *m*/*z* 47. Standard uncertainty $u(r_{AB})$ was estimate using repeatability standard deviation. Procedural blank for nitrate needs to be evaluated. The blank-matching approach can be used [1]. Blank contribution from $Et_3OBF_4$ and be either in the form of $NO_3^-$ and/or $EtONO_2$. An approach to deal with such a contamination is reported in ESM Excel file (Tab: RAW_DATA, Line 283)

## 13. Calculation of isotope dilution result and uncertainty

*Raw data and calculations:*

Find the example in ESM Excel file, Tab: CHAR_HOM, Lines 547-613.
Both nitrite and nitrate were quantified by isotope dilution using a calibration curve fitted with a rational function $y = (a_0 + a_1x) / (1 + a_2x)$ [2]. For isotope dilution calculation and uncertainty evaluation two macro functions were coded: `ResultIDMS_GS` and `ResultIDMS_MC`. The first function estimates uncertainty using error propagation whereas the second is a Monte Carlo method. Details regarding this software can be found in ESM Excel file, Tab: SOFTWARE, Line 236 and the source code is reported in ESM Excel file VBA Module 5.

## 14. Isotopic composition of nitrite and nitrate in sample and primary standard

*Raw data and calculations:*

ESM Excel file, Tab: CHAR_HOM, Line 1082.

*Preparation:*

Seven 2.0 mL aliquots of MOOS-4 and seven 2.0 mL aliquots of a primary standard (nitrite = 0.0776 µg/g, nitrate = 1.06 µg/g) were analyzed without adding the internal standard. No differences between the natural isotopic composition of the two materials were noticed.

## 15. Endogenous presence of EtONO and $EtONO_2$

*Raw data and calculations:*

ESM Excel file, Tab: CHAR_HOM, Line 1074.

*Preparation:*

An aliquot of 2.0 mL of MOOS-4 seawater were transferred into a 10 mL headspace CTC CombiPAL vial and analyzed for nitrite and nitrate (omitting the derivatization with $Et_3OBF_4$). No signals for nitrite and nitrate were detected.

**Paragraph S2.** Spectrophotometric determination of nitrite in seawater

1. **Preparation of the primary standard solutions**

   *Raw data and calculations:*
   ESM Excel file, Tab: CHAR_HOM (for examples see Lines 270-303)

   *Composition:*

| | Target $w(NO_2^-)$ µg/g | Target $w(PO_4^{3-})$ µg/g | Target $w(SiO_2)$ µg/g |
|---|---|---|---|
| Standard 1 | 0.0380 | 0.1316 | 0.2119 |
| Standard 2 | 0.0570 | 0.1973 | 0.3178 |
| Standard 3 | 0.0760 | 0.2631 | 0.4237 |
| Standard 4 | 0.1140 | 0.3947 | 0.6356 |
| Standard 5 | 0.1800 | 0.5262 | 0.8474 |

   *Medium:*
   Low nutrient seawater from OSIL

2. **Sulfanilamide reagent (called NO2-R1)**

   *Preparation:*
   A 5.0 mL volume of concentrated (37%) hydrochloric acid was added to 30 mL of water. The solution was gently mixed and allowed to cool down. An aliquot of 0.5 g of sulfanilamide was added and dissolved. The solution was further diluted with water to a final mass of 50.0 g. This solution was stored in a polyethylene bottle at 4 °C for one month.

3. **N-(1-naphthyl)-ethylenediamine reagent (called NO2-R2)**

   *Preparation:*
   An aliquot of 0.1 g of N-(1-naphthyl)-ethylenediamine dihydrochloride was dissolved in 100 mL of water. This solution was stored in a polyethylene bottle at 4 °C for one month.

4. **Reagent used for baseline correction (called NO2-Acid)**

   *Preparation:*
   A 5.0 mL volume of concentrated (37%) hydrochloric acid was added to 30 mL of water. The solution was gently mixed and allowed to cool down. The solution was further diluted with water to a final mass of 100.0 g. This solution was stored in a polyethylene bottle at room temperature for three months.

## 5. Color development and UV–vis detection

*Preparation:*

A 5 mL volume of the sample (or standard) was mixed with 0.1 mL of NO2-R1. After 5 min, an aliquot of 0.1 mL of NO2-R2 was added. The masses were recorded:

$m_S$ = mass of sample or standard

$m_F$ = $m_S$ + mass of the added reagents

An example of gravimetric preparation data can be found in ESM Excel file, Tab: CHAR_HOM, Lines 361-387.

The reagents blanks were prepared similarly, but their masses were not recorded. Reagent blanks were prepared with both ultrapure water and low nutrient seawater.

The absorbance of all solutions was read after 15 min at 541 nm (5 s integration time, 1 nm spectral bandwidth, 1 cm quartz cuvette). The derivatization was performed in plastic bottles.

## 6. Baseline evaluation and UV–vis detection

*Preparation:*

A 5 mL volume of medium (i.e., seawater samples, ultrapure water, low nutrient seawater) was mixed with 0.2 mL of NO2-Acid. The absorbance of the solution was read after 15 min at 541 nm (5 s integration time, 1 nm spectral bandwidth, 1 cm quartz cuvette). This preparation was performed in plastic bottles. This procedure allowed to evaluate the absorbance of the underivatized matrix within similar conditions employed for color development.

## 7. Data analysis and quantitation

*Raw data and calculations:*

An example of data analysis can be found in ESM Excel file, Tab: CHAR_HOM, Lines 614-650.

*Absorbance corrections:*

Five sequential steps of absorbance correction were performed:

*Baseline correction*: the signal of the underivatized medium (i.e., the absorbance recorded as described at point 6) was subtracted from the analytical signals of blanks, standards, and samples (i.e., the absorbance recorded as described at point 5).

*Blank correction*: the signals of the standards were corrected only for the blank contribution from the low nutrient seawater. The blank arising from the reagents contributed equally to standard and samples, therefore it was not subtracted.

*Gravimetric correction*: the signals of standards and samples were multiplied by the gravimetric factor $m_F/m_S$.

*Density correction*: The signals of standards and samples were divided by the density of the corresponding medium.

*Matrix effect correction*: The signals of the MOOS-4 samples were divided by the α($NO_2^-$) factor. α is the ratio between the slope of the calibration curve obtained within the MOOS-4 medium and the slope of the calibration curve obtained within the low nutrient seawater medium. See ESM Excel file, Tab: CHAR_HOM, Lines 257-268.

*Calibration and uncertainty evaluation:*

A calibration curve was obtained by plotting the nitrite mass fraction of the standards *vs* the corrected absorbance values. The linear model ($y = a_0 + a_1 \cdot x$) was used for quantitation and the uncertainty was evaluated by error propagation. A custom Excel function (`ResultEXLN_GS`) was used to perform this calculation (ESM Excel file, Tab: SOFTWARE, Line176, and Module 4). For verification, also a Monte Carlo method was used with custom Excel function `ResultEXLN_MC` (ESM Excel file, Tab: SOFTWARE, Line176, and Module 4). Results generated using the linear model were also compared against the results generated with the quadratic model ($y = a_0 + a_1 \cdot x + a_2 \cdot x^2$, custom Excel function `ResultEXQD`, ESM Excel file, Tab: SOFTWARE, Line 288, and Module 6).

**Paragraph S3.** Spectrophotometric determination of phosphate in seawater

1. **Preparation of the primary standard solutions**

   *Raw data and calculations:*
   ESM Excel file, Tab: CHAR_HOM (for examples see Lines 270-303)

   *Composition:*

| | Target $w(NO_2^-)$ µg/g | Target $w(PO_4^{3-})$ µg/g | Target $w(SiO_2)$ µg/g |
|---|---|---|---|
| Standard 1 | 0.0380 | 0.1316 | 0.2119 |
| Standard 2 | 0.0570 | 0.1973 | 0.3178 |
| Standard 3 | 0.0760 | 0.2631 | 0.4237 |
| Standard 4 | 0.1140 | 0.3947 | 0.6356 |
| Standard 5 | 0.1800 | 0.5262 | 0.8474 |

   *Medium:*
   Low nutrient seawater from OSIL

2. **Sulfuric acid reagent (called PO4-R1)**
   $[H_2SO_4] \approx 3.8$ mol/L

   *Preparation:*
   A 3.8 M solution of sulfuric acid was prepared by slowly adding 20 mL of concentrated sulfuric acid to 80 mL water. The solution was stored in a polyethylene bottle at room temperature for three months.

3. **Ammonium molybdate reagent (called PO4-R2)**
   [Mo] ≈ 0.268 mol/L

   *Preparation:*
   A portion of 1.42 g of ammonium molybdate - $(NH_4)_6Mo_7O_{24} \cdot 4H_2O$ - was dissolved in 30 mL water. The solution was stored in a polyethylene bottle at room temperature for one month (protected from light).

4. **Antimony potassium tartrate reagent (called PO4-R3)**
   [Sb] ≈ 48.2 mmol/L

   *Preparation:*
   A portion of 0.148 g antimony potassium tartrate hydrate - $C_8H_4K_2O_{12}Sb_2 \cdot xH_2O$ - was dissolved in 10 mL water. The solution was stored in a polyethylene bottle at room temperature for three months.

5. **Phosphate combined reagent (called PO4-MIX)**
   [$H_2SO_4$] ≈ 2.8 mol/L, [Mo] ≈ 53.6 mmol/L, [Sb] ≈ 2.41 mmol/L

   *Preparation:*

   The combined reagent was prepared by mixing 2 mL of PO4-R2, 0.5 mL PO4-R3, and 7.5 mL PO4-R1. The solution was stored in a polyethylene bottle at room temperature and was prepared daily.

6. **Ascorbic acid reagent (called PO4-AA)**
   [$C_6H_8O_6$] ≈ 0.335 mol/L

   *Preparation:*

   A portion of 0.59 g of ascorbic acid was dissolved in 10 mL water. The solution was stored in a polyethylene bottle at 4 °C and it was prepared daily.

7. **Reagent used for baseline correction (called PO4-Acid)**
   [$H_2SO_4$] ≈ 2.8 mol/L, [Sb] ≈ 2.41 mmol/L

   *Preparation:*

   This reagent was prepared by mixing 2 mL of water, 0.5 mL PO4-R3, and 7.5 mL PO4-R1. The solution was stored in a polyethylene bottle at room temperature for three months.

8. **Color development and UV–vis detection**

   *Preparation:*

   A 6 mL volume of the sample (or standard) was mixed with 0.5 mL of PO4-MIX. Then, 0.2 mL of PO4-AA was added. The masses were recorded:
   $m_S$ = mass of sample or standard
   $m_F$ = $m_S$ + mass of the added reagents
   An example of gravimetric preparation data can be found in ESM Excel file, Tab: CHAR_HOM, Lines 392-420.
   The reagents blanks were prepared similarly, but their masses were not recorded. Reagent blanks were prepared with both ultrapure water and low nutrient seawater. The absorbance of all solutions was read after 30 min at 890 nm (5 s integration time, 1 nm spectral bandwidth, 5 cm quartz cuvette). The derivatization was performed in plastic bottles.

9. **Baseline evaluation and UV–vis detection**

   *Preparation:*

   A 6 mL volume of medium (i.e., seawater samples, ultrapure water, low nutrient seawater) was mixed with 0.5 mL of PO4-Acid and with 0.2 mL of PO4-AA. The absorbance of the solution was read after 30 min at 890 nm (5 s integration time, 1 nm spectral bandwidth, 5 cm quartz cuvette). This preparation was performed in plastic bottles. This procedure allowed to evaluate the absorbance of the underivatized matrix within similar conditions employed for color development.

## 10. Data analysis and quantitation

*Raw data and calculations:*

An example of data analysis can be found in ESM Excel file, Tab: CHAR_HOM, Lines 651-689.

*Absorbance corrections:*

Five sequential steps of absorbance correction were performed:

*Baseline correction*: the signal of the underivatized medium (i.e., the absorbance recorded as described at point 9) was subtracted from the analytical signals of blanks, standards, and samples (i.e., the absorbance recorded as described at point 8).

*Blank correction*: the signals of the standards were corrected only for the blank contribution from the low nutrient seawater. The blank arising from the reagents contributed equally to standard and samples, therefore it was not subtracted.

*Gravimetric correction*: the signals of standards and samples were multiplied by the gravimetric factor $m_F/m_S$.

*Density correction*: The signals of standards and samples were divided by the density of the corresponding medium.

*Matrix effect correction*: No matrix effects were observed for phosphate. See ESM Excel file, Tab: CHAR_HOM, Lines 257-268.

*Calibration and uncertainty evaluation:*

A calibration curve was obtained by plotting the phosphate mass fraction of the standards *vs* the corrected absorbance values. The linear model ($y = a_0 + a_1 \cdot x$) was used for quantitation and the uncertainty was evaluated by error propagation. A custom Excel function (`ResultEXLN_GS`) was used to perform this calculation (ESM Excel file, Tab: SOFTWARE, Line176, and Module 4). For verification, also a Monte Carlo method was used with custom Excel function `ResultEXLN_MC` (ESM Excel file, Tab: SOFTWARE, Line176, and Module 4). Results generated using the linear model were also compared against the results generated with the quadratic model ($y = a_0 + a_1 \cdot x + a_2 \cdot x^2$, custom Excel function `ResultEXQD`, ESM Excel file, Tab: SOFTWARE, Line 288, and Module 6).

**Paragraph S4.** Spectrophotometric determination of silicate in seawater

1. **Preparation of the primary standard solutions**

   *Raw data and calculations:*

   ESM Excel file, Tab: CHAR_HOM (for examples see Lines 270-303)

   *Composition:*

| | Target $w(NO_2^-)$ µg/g | Target $w(PO_4^{3-})$ µg/g | Target $w(SiO_2)$ µg/g |
|---|---|---|---|
| Standard 1 | 0.0380 | 0.1316 | 0.2119 |
| Standard 2 | 0.0570 | 0.1973 | 0.3178 |
| Standard 3 | 0.0760 | 0.2631 | 0.4237 |
| Standard 4 | 0.1140 | 0.3947 | 0.6356 |
| Standard 5 | 0.1800 | 0.5262 | 0.8474 |

   *Medium:*

   Low nutrient seawater from OSIL

2. **Sulfuric acid reagent (called Si-R1)**

   $[H_2SO_4] \approx 4.7$ mol/L

   *Preparation:*

   A 4.7 M solution of sulfuric acid was prepared by slowly adding 10 mL of concentrated sulfuric acid to 30 mL water. The solution was stored in a polyethylene bottle at room temperature for three months.

3. **Ammonium molybdate reagent (called Si-R2)**

   [Mo] ≈ 0.36 mol/L

   *Preparation:*

   A portion of 1.27 g ammonium molybdate - $(NH_4)_6Mo_7O_{24} \cdot 4H_2O$ - was dissolved in 10 mL of water. After complete dissolution, 10 mL of Si-R1 was added. The solution was stored in a polyethylene bottle at room temperature for one month (protected from light).

4. **Oxalic acid reagent (called Si-R3)**

   $[H_2C_2O_4 \cdot 2H_2O] \approx 0.79$ mol/L

   *Preparation:*

   A portion of 1.0 g oxalic acid dehydrate was dissolved in 10 mL water. The solution was stored in a polyethylene bottle at room temperature for three months.

5. **Ascorbic acid reagent (called Si-AA)**
   [$C_6H_8O_6$] ≈ 0.16 mol/L

   *Preparation:*
   A portion of 0.28 g ascorbic acid was dissolved in 10 mL water. The solution was stored in a polyethylene bottle at 4 °C and it was prepared daily.

6. **Reagent used for baseline correction (called Si-Acid)**
   [$H_2SO_4$] ≈ 2.4 mol/L

   *Preparation:*
   An aliquot of 10 mL of Si-R1 were added to 10 mL of water. The solution was stored in a polyethylene bottle at room temperature for three months.

7. **Color development and UV–vis detection**

   *Preparation:*
   A 3 mL volume of the sample (or standard) was mixed with 0.12 mL of Si-R2. After 10 min, 0.12 mL of Si-R3 and 0.06 mL of Si-AA were added. The solution was mixed after every addition. Between addition of Si-R3 and Si-AA there was no waiting time. The masses were recorded:
   $m_S$ = mass of sample or standard
   $m_F$ = $m_S$ + mass of the added reagents
   An example of gravimetric preparation data can be found in ESM Excel file, Tab: CHAR_HOM, Lines 460-490.
   The reagents blanks were prepared similarly, but their masses were not recorded. Reagent blanks were prepared with both ultrapure water and low nutrient seawater. The absorbance of all solutions was read after 60 min at 810 nm (5 s integration time, 1 nm spectral bandwidth, 1 cm quartz cuvette). The derivatization was performed in plastic bottles.

8. **Baseline evaluation and UV–vis detection**

   *Preparation:*
   A 3 mL volume of medium (i.e., seawater samples, ultrapure water, low nutrient seawater) was mixed with 0.12 mL of Si-Acid. After 10 min, 0.12 mL of Si-R3 and 0.06 mL of Si-AA were added. The solution was mixed after every addition. Between addition of Si-R3 and Si-AA there was no waiting time.
   The absorbance of the solution was read after 60 min at 810 nm (5 s integration time, 1 nm spectral bandwidth, 1 cm quartz cuvette). This preparation was performed in plastic bottles. This procedure allowed to evaluate the absorbance of the underivatized matrix within similar conditions employed for color development.

9. **Data analysis and quantitation**

   *Raw data and calculations:*
   An example of data analysis can be found in ESM Excel file, Tab: CHAR_HOM, Lines 690-729.

*Absorbance corrections:*

Five sequential steps of absorbance correction were performed:

*Baseline correction*: the signal of the underivatized medium (i.e., the absorbance recorded as described at point 8) was subtracted from the analytical signals of blanks, standards, and samples (i.e., the absorbance recorded as described at point 7).

*Blank correction*: the signals of the standards were corrected only for the blank contribution from the low nutrient seawater. The blank arising from the reagents contributed equally to standard and samples, therefore it was not subtracted.

*Gravimetric correction*: the signals of standards and samples were multiplied by the gravimetric factor $m_F/m_S$.

*Density correction*: The signals of standards and samples were divided by the density of the corresponding medium.

*Matrix effect correction*: No matrix effects were observed for silicate. See ESM Excel file, Tab: CHAR_HOM, Lines 257-268.

*Calibration and uncertainty evaluation:*

A calibration curve was obtained by plotting the silicate mass fraction of the standards *vs* the corrected absorbance values. The linear model ($y = a_0 + a_1 \cdot x$) was used for quantitation and the uncertainty was evaluated by error propagation. A custom Excel function (`ResultEXLN_GS`) was used to perform this calculation (ESM Excel file, Tab: SOFTWARE, Line176, and Module 4). For verification, also a Monte Carlo method was used with custom Excel function `ResultEXLN_MC` (ESM Excel file, Tab: SOFTWARE, Line176, and Module 4). Results generated using the linear model were also compared against the results generated with the quadratic model ($y = a_0 + a_1 \cdot x + a_2 \cdot x^2$, custom Excel function `ResultEXQD`, ESM Excel file, Tab: SOFTWARE, Line 288, and Module 6).

**Paragraph S5.** HPLC–ICP–MS determination of silicate in seawater

## 1. Preparation of the internal standard solution

*Composition:*

$w(^{30}SiO_2)$ ~ 0.4 µg/g

*Medium:*

Diluted HCl (prepared by adding 0.2 mL of 36 % HCl into 2 L of ultrapure water)

## 2. Preparation of the primary standard solutions

*Raw data and calculations:*

ESM Excel file, Tab: CHAR_HOM (for examples see Lines 1137-1139)

*Composition:*

| | Target $w(SiO_2)$ µg/g |
|---|---|
| Standard 1 | 0.21 |
| Standard 2 | 0.42 |
| Standard 3 | 0.82 |

*Medium:*

Ultrapure water

## 3. Preparation of the blends for isotope dilution quantitation

*Raw data and calculations:*

ESM Excel file, Tab: CHAR_HOM, Lines 1141-1158

*Preparation:*

0.5 mL of sample (or primary standard solution) was transferred into a polypropylene HPLC vial followed by 0.5 mL of internal standard solution. The masses of the solutions were recorded. The blends were thoroughly mixed.

## 4. Mobile phase

*Preparation:*

Diluted HCl solution was used as mobile phase and it was prepared in a polypropylene HPLC bottle by adding 0.1 mL of 36 % HCl into 1 L of ultrapure water.

## 5. HPLC–ICP–MS method for detection of silicate

*Instrumental conditions for HPCL:*

| | |
|---|---|
| Column: | ICE-AS1 (9 mm id x 250 mm) |
| Mobile phase: | Diluted HCl |
| Elution mode: | Isocratic |
| Flow rate: | 0.25 mL/min |
| Injection volume: | 25 µL |

*Instrumental conditions for ICP–MS:*

| | |
|---|---|
| Monitored *m*/*z* (SIM mode): | 28, 30 |
| Dwell time: | 0.3 s |
| Detector: | forced analogue |
| Rection gas (flow rate): | $O_2$, (20 %) |

## 6. Calculation of isotope ratio and blank correction

*Raw data and calculations:*

Find the example in ESM Excel file, Tab: RAW_DATA, Line 870.

The isotope ratio $r_{AB}$ for silicate is the ratio between the chromatographic area at *m*/*z* 28 and at *m*/*z* 30. Standard uncertainty $u(r_{AB})$ was estimate using repeatability standard deviation. Procedural blank for silicate is not usually significant when labware made of glass is avoided during the preparation of samples and standards.

## 7. Calculation of isotope dilution result and uncertainty

*Raw data and calculations:*

Both nitrite and nitrate were quantified by isotope dilution using a calibration curve fitted with a rational function $y = (a_0 + a_1x) / (1 + a_2x)$ [2]. For isotope dilution calculation and uncertainty evaluation two macro functions were coded: `ResultIDMS_GS` and `ResultIDMS_MC`. The first function estimates uncertainty using error propagation whereas the second is a Monte Carlo method. Details regarding this software can be found in ESM Excel file, Tab: SOFTWARE, Line 236 and the source code is reported in ESM Excel file VBA Module 5.

**Paragraph S6.** Headspace GC–MS standardization of nitrite (67276 MilliporeSigma)

1. **Sulfuric acid reagent**
   [$H_2SO_4$] ≈ 0.75 N (≈ 0.375 mol/L)

   *Preparation:*
   A 0.75 N solution of sulfuric acid was prepared by slowly adding 2.04 mL of concentrated sulfuric acid to 97.96 mL water. The solution was stored in a polyethylene bottle at room temperature for three months.

2. **Permanganate reagent**
   [$KMnO_4$] ≈ 0.1 N (≈ 0.02 mol/L)

   *Preparation:*
   A portion of 0.06 g of $KMnO_4$ was dissolved in ultrapure water to a final mass of 20.0 g of solution. The solution was stored in an amber glass vial at room temperature for three months.

3. **Sulfamic acid reagent**
   $NH_2SO_3H$ ≈ 1 % w/w

   *Preparation:*
   A portion of 0.3 g of sulfamic acid was dissolved in 29.7 g of ultrapure water. The solution was kept in a plastic bottle at 4 °C for no longer than 1 month.

4. **Preparation of the internal standard solution of $^{15}NO_3^-$**

   *Raw data and calculations:*
   ESM Excel file, Tab: RND, Lines 703-709

   *Composition:*
   $w(^{15}NO_3^-)$ ~ 96.24 µg/g

   *Medium:*
   Ultrapure water

   *Solution ID:*
   NO3-IntST

5. **Dilution of the MilliporeSigma nitrite standard (p/n 67276, Lot BCCK5026) for nitrite testing**

   *Raw data and calculations:*
   ESM Excel file, Tab: RND, Lines 712-731

   *Composition:*
   $w(NO_2^-)$ = 75.6 (0.2) µg/g

   *Medium:*
   Ultrapure water

   *Solution ID:*
   NO2-SAMPLE

6. **Dilution of the MilliporeSigma nitrite standard (p/n 67276, Lot BCCK5026) for testing the residual nitrate after nitrite elimination**

   *Raw data and calculations:*
   ESM Excel file, Tab: RND, Lines 734-743

   *Preparation:*
   The following solutions were sequentially mixed:
   *i*. MilliporeSigma nitrite standard (p/n 67276, Lot BCCK5026): 0.7458 g
   *ii*. 1 % sulfamic acid (point 3): 3.0 g
   *iii*. ultrapure water: 6.3855 g

   *Solution ID:*
   NO2-SAMPLE-SULF

7. **Blank of the sulfamic acid solution**

   *Raw data and calculations:*
   ESM Excel file, Tab: RND, Lines 746-754

   *Preparation:*
   The following solutions were sequentially mixed:
   *i*. 1 % sulfamic acid (point 3): 3.0 g
   *ii*. ultrapure water: 6.9 g

   *Solution ID:*
   BLANK-SULF

8. **Preparation of the primary standard solutions of nitrate from NIST SRM 3185**

   *Raw data and calculations:*
   ESM Excel file, Tab: RND, Lines 757-764

   *Composition:*

| Solution ID | $w(NO_3^-)$ µg/g |
|---|---|
| ST1 | 50.16 |
| ST2 | 101.5 |
| ST3 | 206.3 |

   *Medium:*
   Ultrapure water

9. **Preparation of the blends for isotope dilution quantitation**

   *Raw data and calculations:*
   ESM Excel file, Tab: RND, Lines 767-788

   *Preparation:*
   An aliquot of 1.0 mL of sample (or primary standard solution or blank) was transferred into a plastic bottle along with 1.0 mL of internal standard solution

(NO3-IntST). The masses of the solutions were recorded. The blends were thoroughly mixed.

**10. Oxidation of nitrite to nitrate**

*Preparation:*

An aliquot of 1.0 mL of 0.75 N $H_2SO_4$ along with 0.1 mL of 0.1 N potassium permanganate were transferred in a 4 mL glass vial. 11 vials were prepared in this way, capped and incubated at 40 °C for 15 min. At this point, aliquots of 0.2 mL of the blends prepared at point 9 were transferred (by immersing the pipette tip inside the permanganate solution) in each of these 11 vials with the exception of the blends containing sulfamic acid. The solutions were mixed and allowed to react for 10 min. Three replicates of the sample (NO2-SAMPLE), five calibration standards (ST1, triplicate of ST2, ST3) and three procedural blanks (ultrapure water) were prepared in this way. This preparation was meant to establish if the oxidation $NO_2^-$ → $NO_3^-$ was quantitative and to quantify the resulting nitrate by high-precision isotope dilution.

**11. Dilution of the blends containing sulfamic acid**

*Preparation:*

An aliquot of 1.1 mL of ultrapure water was transferred in a 4 mL glass vial. 6 vials were prepared in this way, capped and incubated at 40 °C for 15 min. At this point, aliquots of 0.2 mL of the blends containing sulfamic acid (point 9) were transferred in each of these 6 vials. The solutions were mixed and allowed to react for 10 min. Three replicates of the sample (NO2-SAMPLE-SULF), and three replicates of the blank (BLANK-SULF) were prepared. This preparation was meant to assess whether the original nitrite standard solution (MilliporeSigma nitrite, p/n 67276, Lot BCCK5026) contained any nitrate impurities.

**12. Derivatization chemistry for the determination of nitrate**

*Preparation:*

A 0.5 mL volume of the solutions prepared at points 10 and 11 was transferred into a 10 mL headspace vial suitable for CTC CombiPAL autosampler along with 1.5 mL of ultrapure water. At this point 0.05 mL of triethyloxonium tetrafluoroborate aqueous solution was added (see point 13) and mixed. After 30 min, the mixture was analyzed by headspace GC–MS.

**13. Triethyloxonium tetrafluoroborate aqueous solution**

*Preparation:*

A portion of 1.0 g of $Et_3OBF_4$ was transferred into a PFA vial and an aliquot of 1.0 mL of ultrapure water previously cooled to 4 °C was added. The $Et_3OBF_4$ was quickly dissolved and used within 5 min. Since $Et_3OBF_4$ is unstable in water, leftovers of this solution were discarded.

*Safety precaution:*

$Et_3OBF_4$ is a strong alkylating agent which needs to be handled accordingly to its SDS. All manipulations of the reagent should be performed under a fume hood wearing suitable PPE. $Et_3OBF_4$ is stored at −20 °C protected from light.

## 14. Headspace GC–MS method for detection of nitrite and nitrate (EtONO and $EtONO_2$)

*Instrumental conditions for CTC headspace incubation and sampling:*

| | |
|---|---|
| Injection volume: | 500 µL |
| Syringe size: | 2.5 mL for headspace |
| Incubation temperature: | 60 °C |
| Incubation time: | 2 min |
| Syringe temperature: | 70 °C |
| Agitator speed: | 500 rpm |
| Fill speed: | 100 µl/s |
| Fill strokes: | 3 |
| Pullup delay: | 1000 ms |
| Injection speed: | 500 µl/s |
| Pre inject delay: | 500 ms |
| Post inject delay: | 500 ms |
| Flush time: | 5 min |
| GC runtime: | 12 min |

*Instrumental conditions for GC separation:*

*OVEN*

| | |
|---|---|
| Equilibration time: | 0.5 min |
| Initial temp: | 30 °C |
| Isotherm time: | 2.5 min |
| Ramp 1: | 20 °C/min up to 140 °C |
| isotherm time: | 0.0 min |
| Run time: | 8.00 min |

*FRONT INLET (SPLIT/SPLITLESS)*

| | |
|---|---|
| Mode: | Split |
| Initial temp: | 120 °C |
| Liner type: | 1 mm strait |
| Split ratio: | 7:1 |
| Split flow: | 6.9 mL/min |
| Total flow: | 11.2 mL/min |
| Gas saver: | On |
| Saver flow: | 20.0 mL/min |
| Saver time: | 2.00 min |
| Gas type: | Helium |

*COLUMN*

| | |
|---|---|
| Capillary column: | DB-624 (6%-cyanopropyl-phenyl-94%-dimethyl polysiloxane) |

| | |
|---|---|
| Model number: | J&W 122-1334 |
| Nominal length: | 30 m |
| Nominal diameter: | 250.00 µm |
| Nominal film thickness: | 1.40 µm |
| Mode: | constant flow |
| Initial flow: | 1.0 mL/min |
| MSD transfer line temp: | 220 °C |

*Instrumental conditions for MS detection:*

| | |
|---|---|
| Solvent delay: | 2.1 min |
| EMV mode: | Gain Factor |
| Gain factor: | 1.0 |
| Resulting EM: | 1118 V |
| Segment 1 (nitrite): | From 2.1 min to 3.0 min |
| SIM parameters ion 1: | *m*/*z* 31 (dwell time 50 ms) |
| SIM parameters ion 2: | *m*/*z* 32 (dwell time 50 ms) |
| SIM parameters ion 3: | *m*/*z* 45 (dwell time 50 ms) |
| Segment 2 (nitrate): | From 3.0 min to 7.0 min |
| SIM parameters ion 1: | *m*/*z* 46 (dwell time 50 ms) |
| SIM parameters ion 2: | *m*/*z* 47 (dwell time 50 ms) |
| MS source temp: | 150 °C |
| MS quad temp: | 150 °C |
| Timed events: | MS OFF at 3.0 min;<br>MS ON at 5.0 min;<br>MS OFF at 7.0 min |

*Retention time and raw data:*

Under this program, nitrite (under the form of EtONO) elutes at 2.46 min whereas nitrate (under the form of $EtONO_2$) elutes at 5.8 min. The integration of the signals was obtained using the Agilent MassHunter software (B.06.00, Built 6.0.633.0, Agile integrator, 2012). Areas at *m*/*z* 45 for nitrite and *m*/*z* 46 and *m*/*z* 47 for nitrate were exported in ESM Excel file, Tab: RAW_DATA, Lines 142-240.

## 15. Verify quantitative conversion of nitrite by permanganate

*Raw data and calculations:*

Find ESM Excel file, Tab: RND, Lines 830-839.

*Data analysis:*

The nitrite signal from ST2 (nitrate standard with no nitrite, $n$ = 3) was compared with the nitrite signal of NO2-SAMPLE (nitrite standard reacted with permanganate, $n$ = 3). No difference was observed, hence the reaction between nitrite and permanganate was quantitative.

## 16. Detect eventual nitrate contamination in the nitrite standard (MilliporeSigma, p/n 67276, Lot BCCK5026)

*Raw data and calculations:*

Find ESM Excel file, Tab: RND, Lines 847-859.

*Data analysis:*

The nitrate signal from BLANK-SULF (blank solution reacted with sulfamic acid) was compared with the nitrate signal of NO2-SAMPLE-SULF (nitrite standard reacted with sulfamic acid). No detectable contamination of nitrate was observed for the nitrite standard.

**17. Calculation of nitrate isotope ratio and blank correction**

*Raw data and calculations:*

*Nitrate*: find the example in ESM Excel file, Tab: RAW_DATA, Line 142-240.

The isotope ratio $r_{AB}$ for nitrate is the ratio between the chromatographic area at $m/z$ 46 and at $m/z$ 47. Standard uncertainty $u(r_{AB})$ was estimate using repeatability standard deviation. Procedural blank contribution (<0.3 %) was corrected using the blank-matching approach [1].

**18. Quantitation of nitrate originated from nitrite after oxidation by permanganate**

*Raw data and calculations:*

Find the example in ESM Excel file, Tab: CHAR_HOM, Lines 862-884.

Nitrate was quantified by isotope dilution using a calibration curve fitted with a rational function $y = (a_0 + a_1x) / (1 + a_2x)$ [2]. Isotope dilution results and uncertainty (error propagation) were calculate using the custom macro function named `ResultIDMS_GS`. Details regarding this software can be found in ESM Excel file, Tab: SOFTWARE, Line 236 and the source code is reported in ESM Excel file VBA Module 5.

**19. Calculation of the nitrite content in the nitrite standard (MilliporeSigma, p/n 67276, Lot BCCK5026)**

*Raw data and calculations:*

Find the example in ESM Excel file, Tab: CHAR_HOM, Lines 887-892.

The nitrate mass fraction measured at point 18 was converted into nitrite mass fraction and the dilution factor was applied in order to evaluate the mass fraction of nitrite in the original standard.

**20. Isotopic composition of nitrate in sample and primary standard**

*Raw data and calculations:*

ESM Excel file, Tab: CHAR_HOM, Lines 809-825.

*Preparation:*

Seven aliquots of NO2-SAMPLE-SULF and seven aliquots of ST2 where were analyzed as described at points 10 to 14 without adding the internal standard NO3-IntST. No difference between the natural isotopic composition of the two materials was noticed.

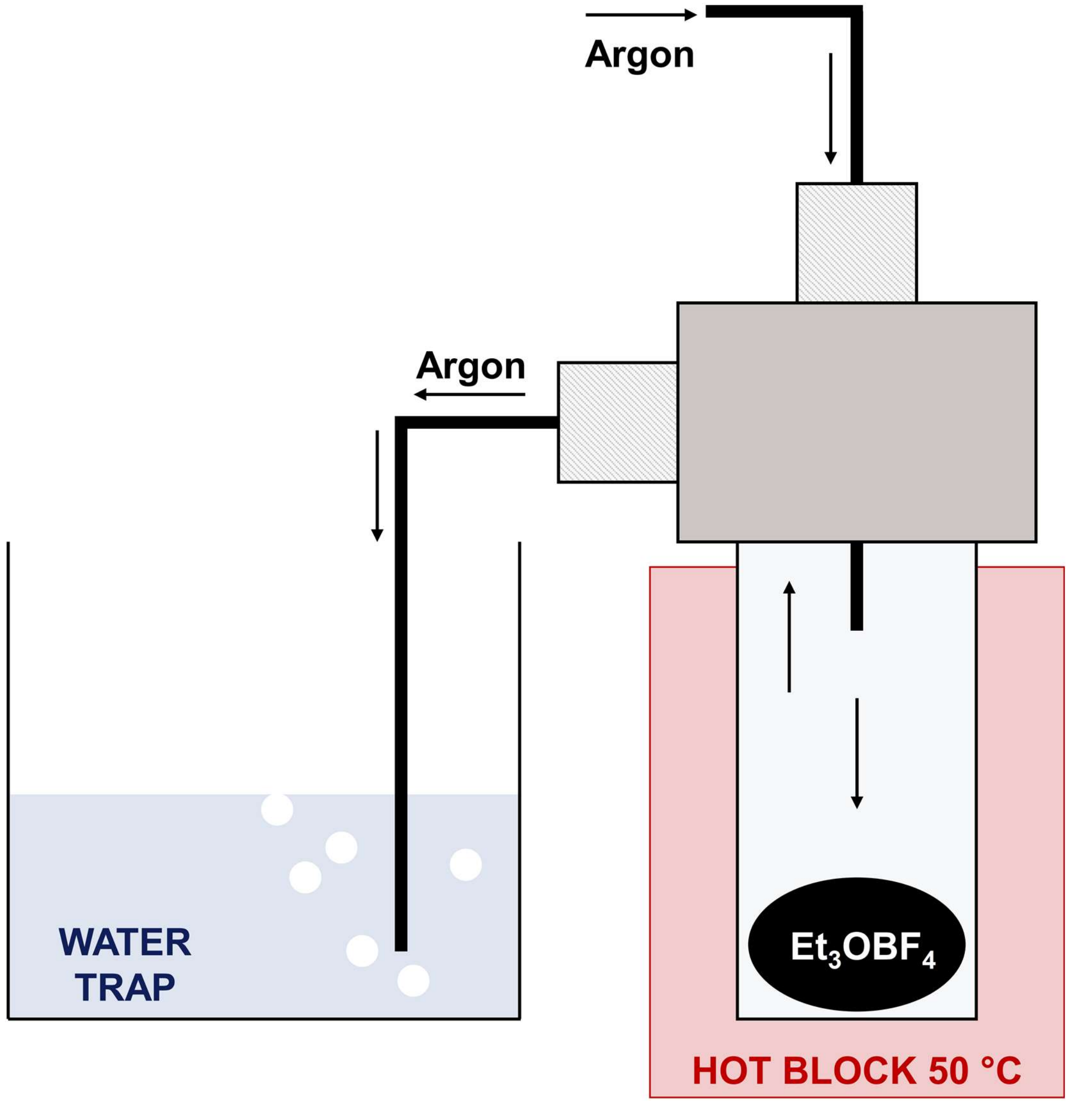


**Figure S1** Manifold for the purification of triethyloxonium tetrafluoroborate

Commercial $Et_3OBF_4$ can be flushed at 50 °C for reducing the level of nitrate contamination (see Paragraph S1). Under these conditions, nitrate is alkylated and removed under the form of volatile $EtONO_2$.

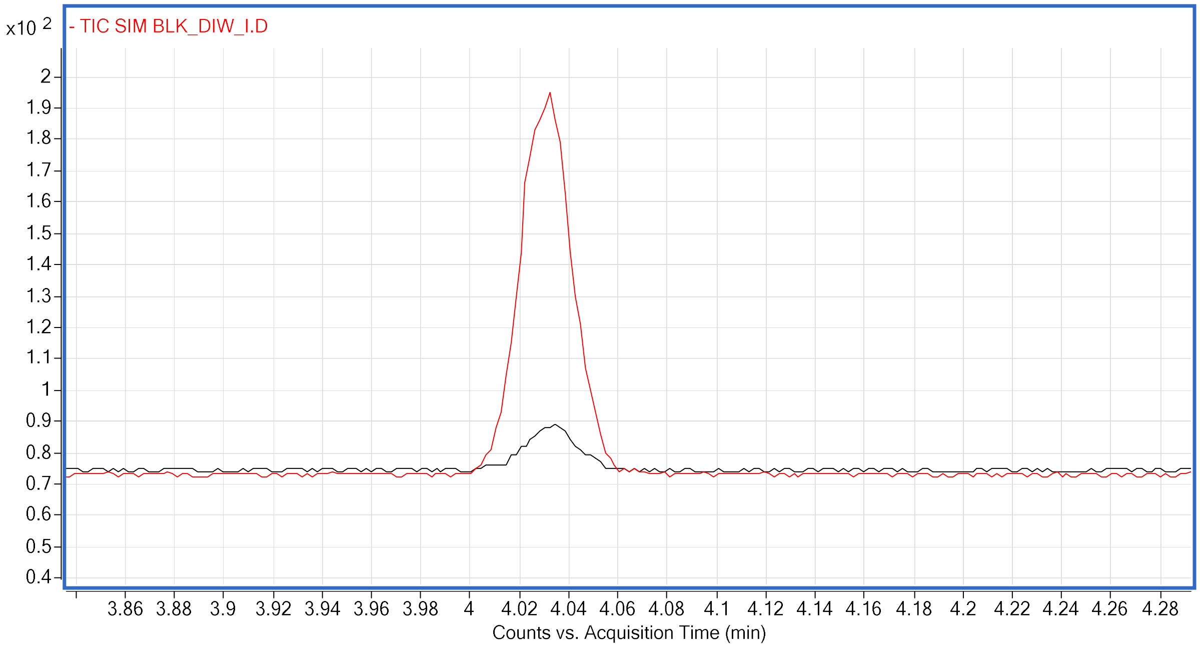


**Figure S2** Purification of triethyloxonium tetrafluoroborate

See Paragraph S1 and Fig. S1. Red: nitrate signal *before* $Et_3OBF_4$ purification. Black: nitrate signal *after* $Et_3OBF_4$ purification.

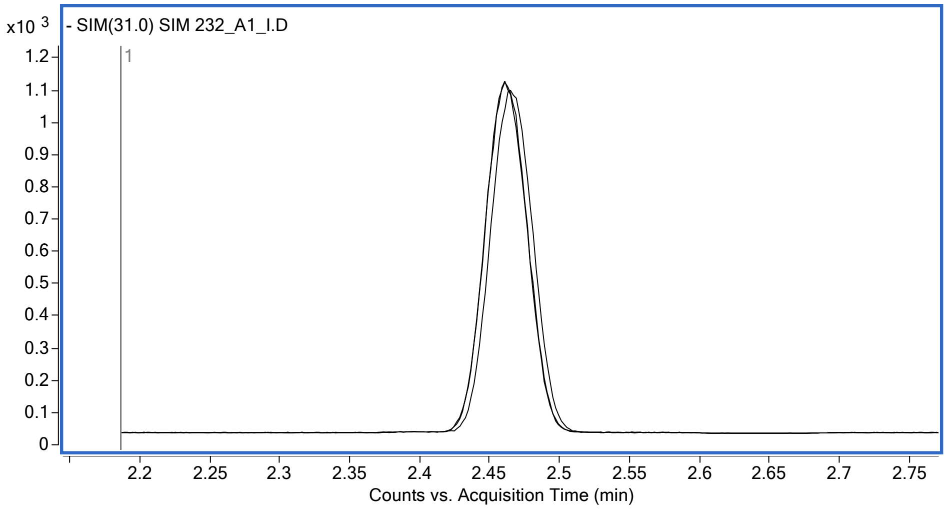


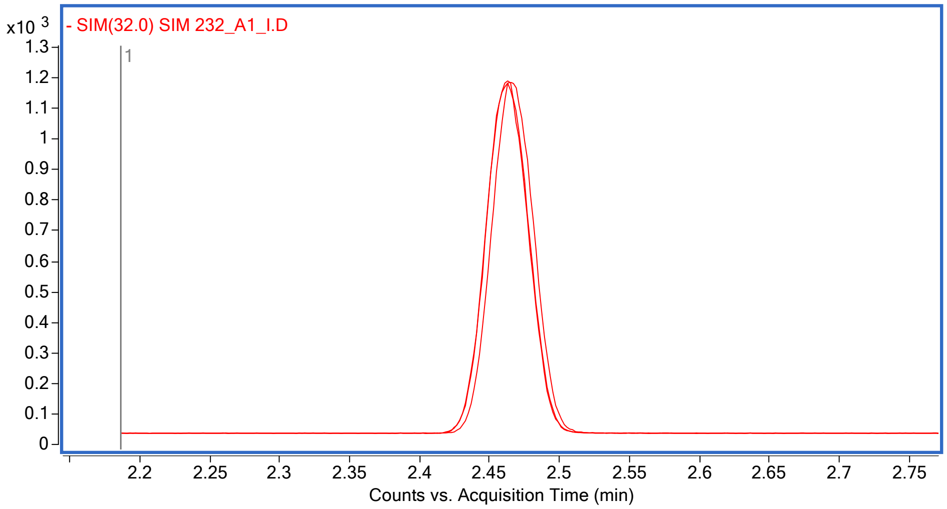


**Figure S3** GC–MS chromatograms of nitrite in MOOS-4

Overlaid signals obtained from three independent measurements of MOOS-4. Top: analytical signal at *m*/*z* 31 (ion fragment $H^{14}NO^{-}$). Bottom: signal of the internal standard at m/z 32 (ion fragment $H^{15}NO^{-}$).

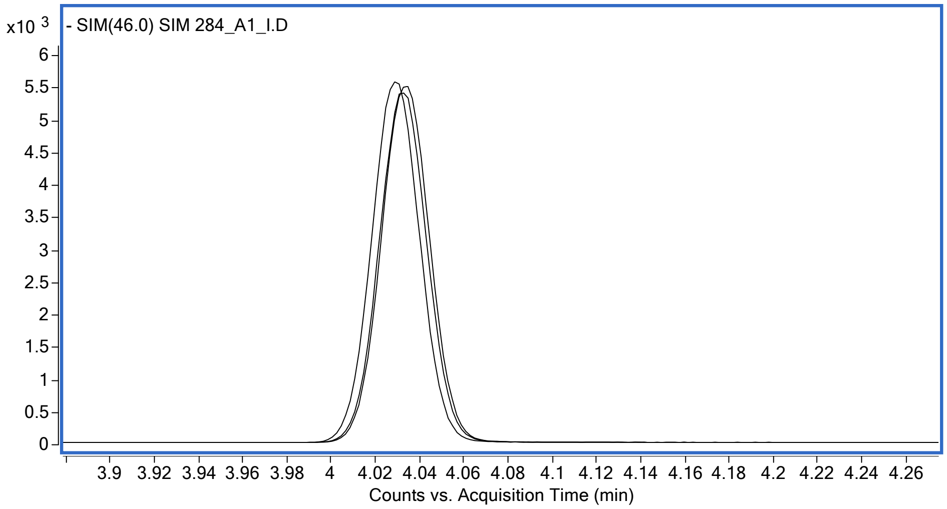


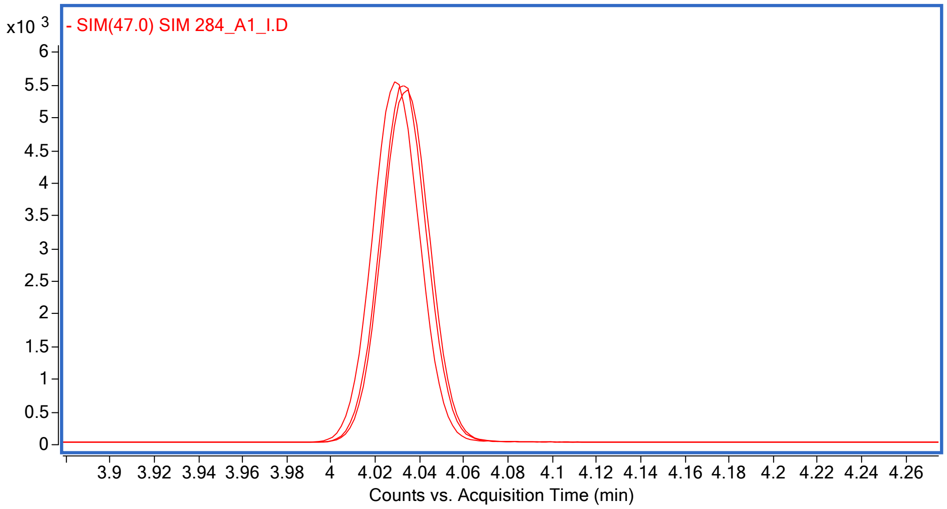


**Figure S4** GC–MS chromatograms of nitrate in MOOS-4

Overlaid signals obtained from three independent measurements of MOOS-4. Top: analytical signal at *m*/*z* 46 (ion fragment $^{14}NO_2^-$). Bottom: signal of the internal standard at m/z 47 (ion fragment $^{15}NO_2^-$).

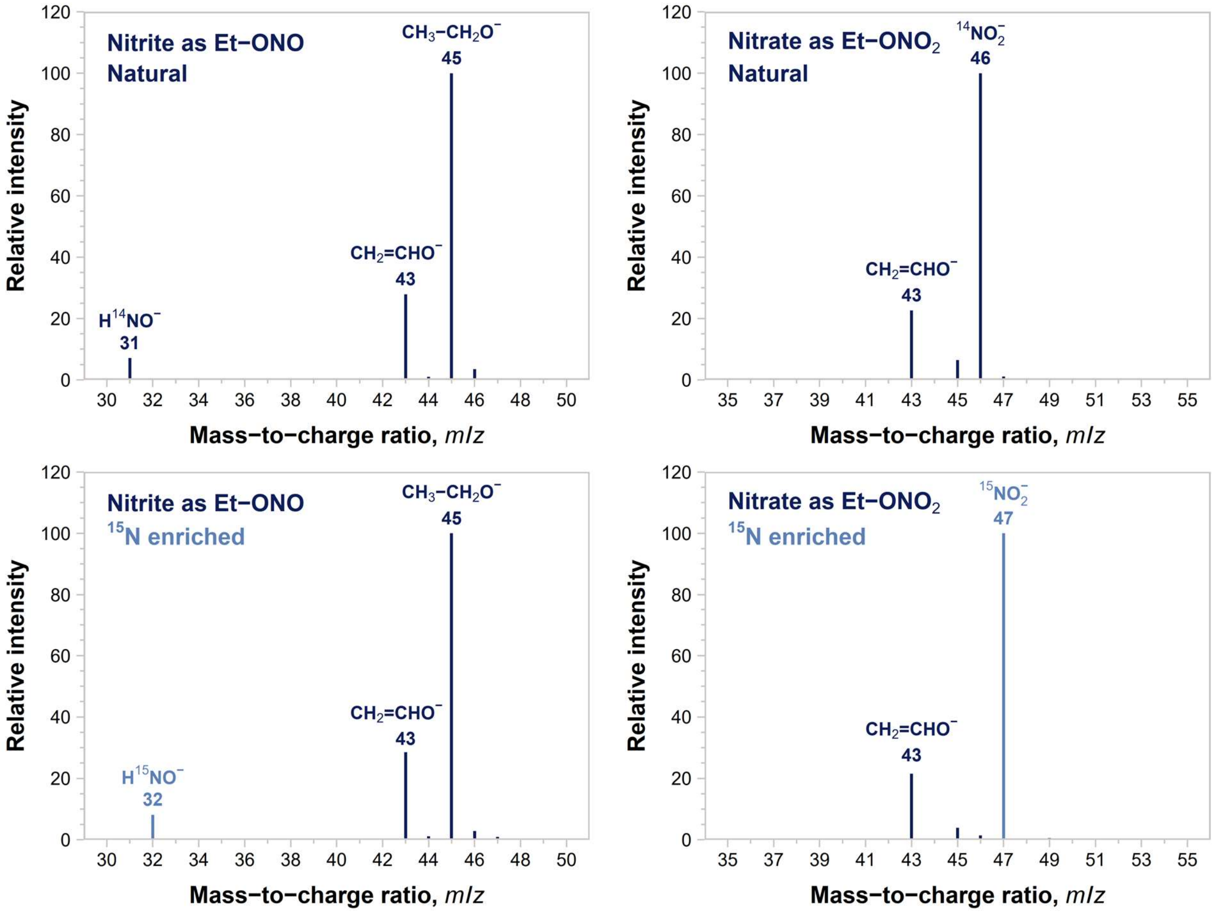


**Figure S5** NCI mass spectra of nitrite and nitrate ethyl-derivatives

For isotope dilution quantitation purposes, signals at *m*/*z* 31 and 32 were monitored for nitrite, whereas signals at *m*/*z* 46 and 47 were monitored for nitrate

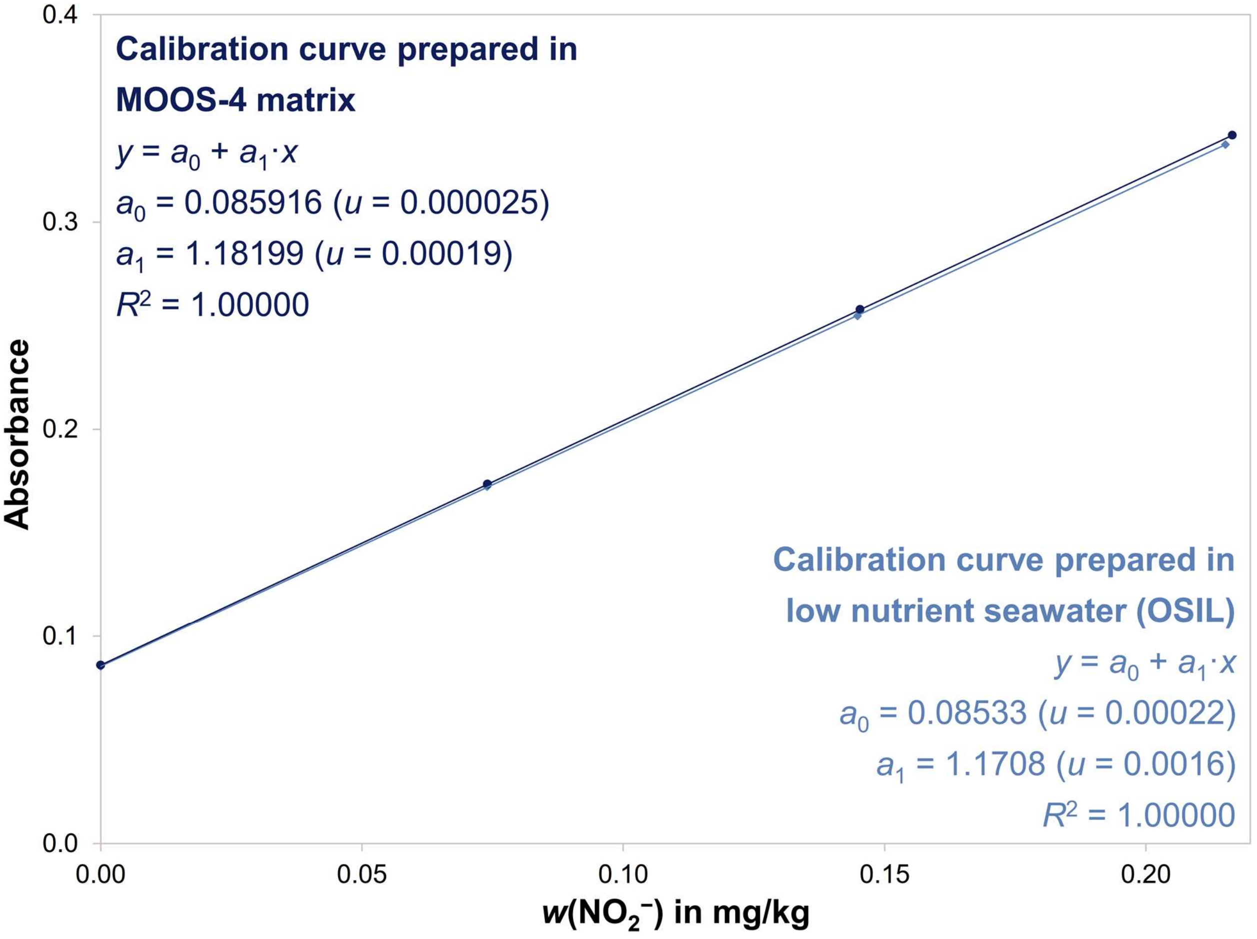


**Figure S6** Determination of nitrite by spectrophotometry: rotational matrix effect

The dark blue line is the calibration curve prepared within the MOOS-4 sample matrix whereas the light blue line is the calibration curve prepared within the low nutrient seawater matrix from OSIL. The ratio between the slopes is proportional to the (small) matrix effect (ESM Excel file, Tab: CHAR_HOM, Line 257)

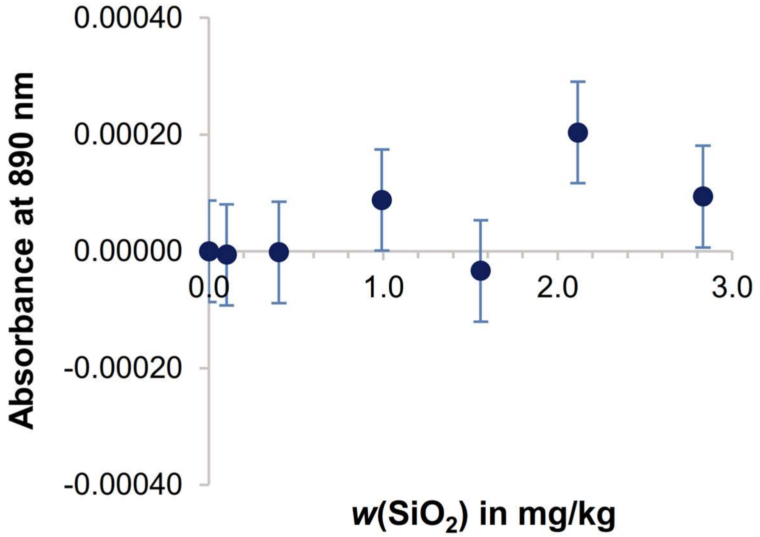


**Figure S7** Silicate interference on phosphate signal at 890 nm

No interference on phosphate signal was observed with 0.0-2.8 mg/kg $SiO_2$ (ESM Excel file, Tab: RND, Line 1654)

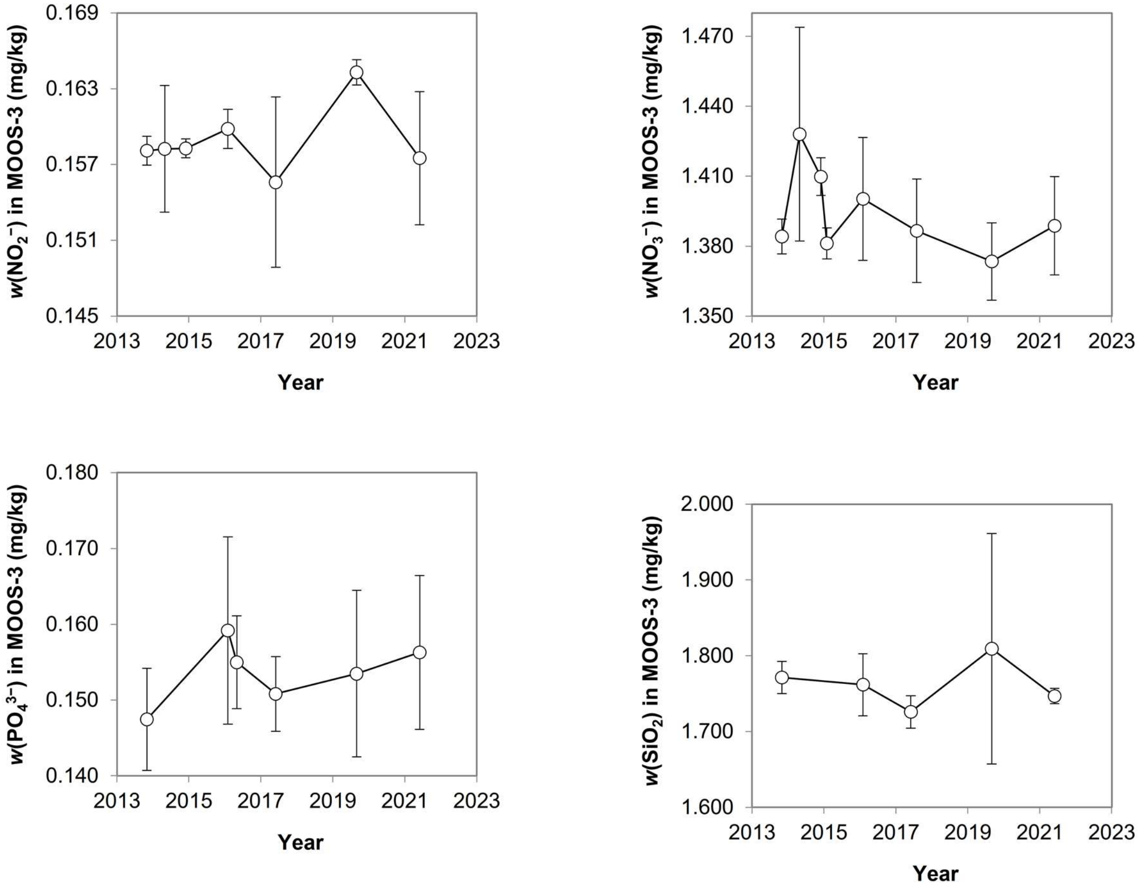


**Figure S8** Long-term stability study on MOOS-3

See also ESM Excel file, Tab: LONG, Lines 9-65. Within the measurement uncertainty, the nutrient composition in previous MOOS-3 seawater CRM was stable.

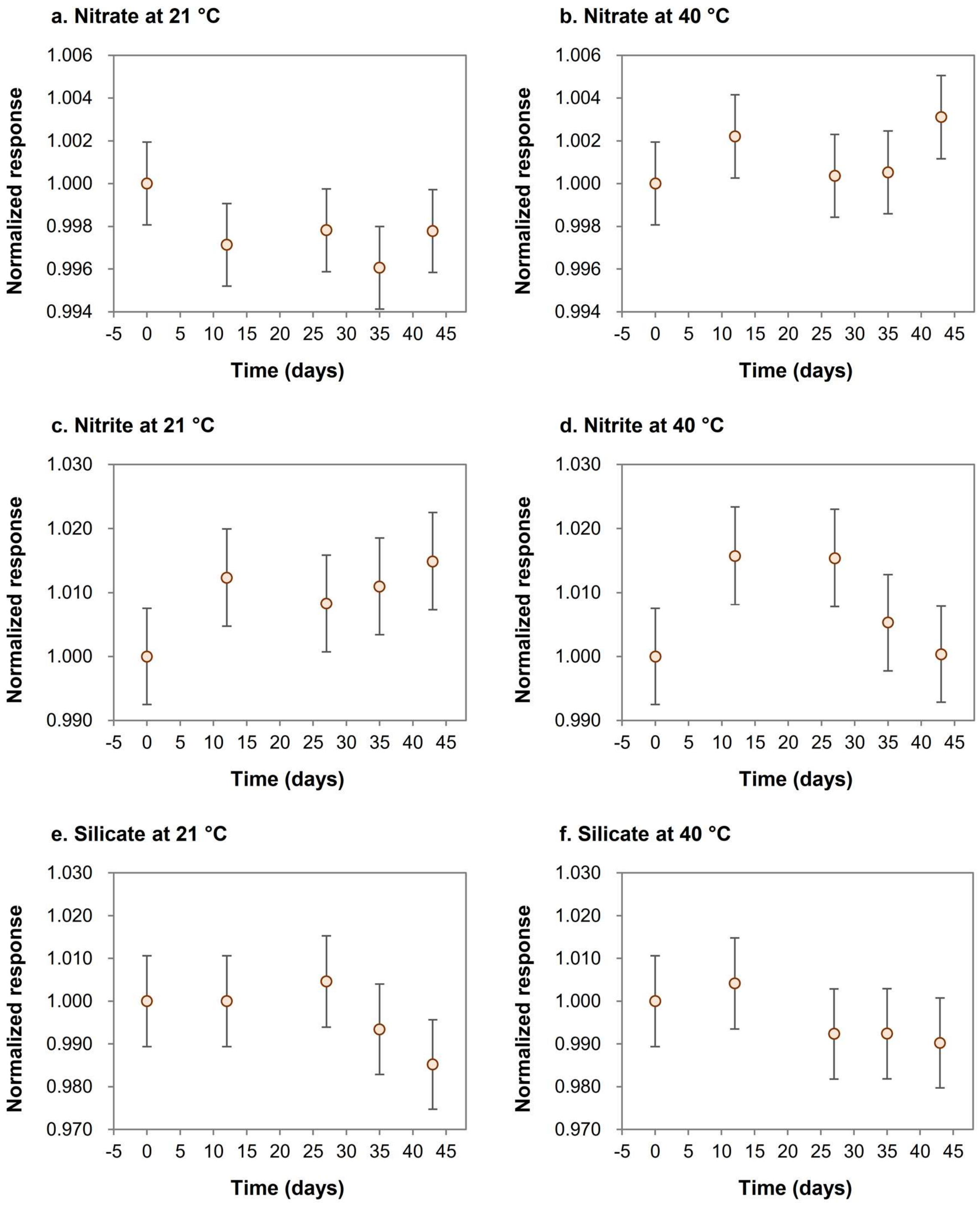


**Figure S9** Accelerated stability study for nitrate, nitrite and silicate in MOOS-4

See also ESM Excel file, Tab: SHORT_HOM. No trends were observed for these analytes at elevated temperature. Each unit was analyzed two times and its response was normalized to the reference units kept at 4 °C.

**References**

1. Pagliano E, Mester Z, Meija J. Blank correction in isotope dilution. Anal Chem. 2015;87(21):10724-7. doi: 10.1021/acs.analchem.5b02924.

2. Pagliano E, Mester Z, Meija J. Calibration graphs in isotope dilution mass spectrometry. Anal Chim Acta. 2015;896:63-7. doi: 10.1016/j.aca.2015.09.020.
